# *De novo* assembly, transcriptome characterization, lignin accumulation, and anatomic characteristics: novel insights into lignin biosynthesis during celery leaf development

**DOI:** 10.1038/srep08259

**Published:** 2015-02-05

**Authors:** Xiao-Ling Jia, Guang-Long Wang, Fei Xiong, Xu-Run Yu, Zhi-Sheng Xu, Feng Wang, Ai-Sheng Xiong

**Affiliations:** 1 State Key Laboratory of Crop Genetics and Germplasm Enhancement, College of Horticulture, Nanjing Agricultural University, Nanjing, 210095, China; 2 Key Laboratories of Crop Genetics and Physiology of the Jiangsu Province and Plant Functional Genomics of the Ministry of Education, Yangzhou University, Yangzhou 225009, China

## Abstract

Celery of the family Apiaceae is a biennial herb that is cultivated and consumed worldwide. Lignin is essential for cell wall structural integrity, stem strength, water transport, mechanical support, and plant pathogen defense. This study discussed the mechanism of lignin formation at different stages of celery development. The transcriptome profile, lignin distribution, anatomical characteristics, and expression profile of leaves at three stages were analyzed. Regulating lignin synthesis in celery growth development has a significant economic value. Celery leaves at three stages were collected, and Illumina paired-end sequencing technology was used to analyze large-scale transcriptome sequences. From Stage 1 to 3, the collenchyma and vascular bundles in the petioles and leaf blades thickened and expanded, whereas the phloem and the xylem extensively developed. Spongy and palisade mesophyll tissues further developed and were tightly arranged. Lignin accumulation increased in the petioles and the mesophyll (palisade and spongy), and the xylem showed strong lignification. Lignin accumulation in different tissues and at different stages of celery development coincides with the anatomic characteristics and transcript levels of genes involved in lignin biosynthesis. Identifying the genes that encode lignin biosynthesis-related enzymes accompanied by lignin distribution may help elucidate the regulatory mechanisms of lignin biosynthesis in celery.

Dietary fiber is the part of plant material resistant to enzymatic digestion, and has a positive effect on improved health status since the consumption is related to decrease the risk of several diseases, such as coronary heart disease and cancer^1^. It includes cellulose, hemicellulose, lignin, oligosaccharides, pectins, gums and waxes^2^. Lignin is an important component of dietary fiber, and naturally present in fruits and vegetables. Lignin is also the important component of plant's cell wall. During plant growth and development, increasing lignin and cellulose deposit in the cell walls (especially in the xylem). With the increasing lignin concentration, cell walls thickness increases and plant tissues become lignified, which affect the taste in vegetable.

Celery (*Apium graveolens* L.), rich in dietary fiber, of the family Apiaceae is a biennial herb that originated from the Mediterranean basin and is currently cultivated and consumed worldwide^3^. This biennial herb was initially grown for medicinal use and then consumed in the 17^th^ century in Italy, France, and England^4^. Celery is low in calorie and rich in carotenoids, flavonoids, volatile oils, and fiber^5^. ‘Ventura', a celery variety with thick glossy leaves, originated from the United States and was later introduced to China. This cultivar is well known for its high disease resistance and high yield.

Next-generation sequencing (NGS) technologies, such as Roche/454 and Illumina High-Seq, can generate high-throughput reads at a relatively low cost. NGS technologies are powerful for *de novo* sequencing, genome resequencing, and whole genome or transcriptome analysis^6^. Sequences obtained contain abundant functional information and essentially perform in revealing the molecular mechanism of functional genes^7^,^8^. NGS technologies present an efficient and economic choice for characterizing nonmodel organisms, such as celery, without a reference genome^9^. Extensive *de novo* genomic or transcriptomic sequences of *A. graveolens* are valuable resources for gene discovery, molecular marker development, gene localization, comparative genomics, etc.^10^,^11^.

The aromatic heteropolymer lignin is the second most abundant biopolymer in secondarily thickened plant cell walls after cellulose. In particular, lignin comprises up to 30% of the total plant biomass^12^. Lignin polymers are primarily derived from the monolignols *p*-hydroxyphenyl (H), guaiacyl (G), and syringyl (S), which are formed by the dehydrogenation of the hydroxycinnamyl alcohols *p*-coumaryl, coniferyl, and sinapyl, respectively^13^. Lignin is predominantly deposited in secondarily thickened cell walls of vascular plants and plays important roles in cell wall structural integrity, stem strength, water transport, mechanical support, and plant pathogen defense^14^. Thus, studying the effects of lignin amount and composition on end-use properties and understanding the regulatory factors of lignin biosynthesis are indispensable to improve plant quality.

The main building blocks of lignin are monolignols (*p*-hydroxyphenyl, guaiacyl, and syringyl). Numerous studies have elucidated the mechanism of lignin formation and have identified the corresponding enzymes and genes responsible for lignin biosynthesis^15^. Monolignols are derived from phenylalanine through the phenylpropanoid pathway^16^. Cinnamic acid is initially formed by phenylalanine (PAL) deamination, followed by a series of hydroxylation (C4H, C3H, F5H, 4CL, and HCT), O-methylation (COMT and CCoAOMT), and reduction reactions (CCR and CAD). Lignin is finally formed by dehydrogenation polymerization (POD or LAC) after the synthesis of monolignols. The methoxylation levels of the three monolignols determine the amount and composition of lignin^17^.

Transcriptomes and microRNAs of three celery cultivars, namely, *A. graveolens* cvs ‘Liuhe Huangxinqin', ‘Jinnan Shiqin', and ‘Ventura' are involved in the temperature stress response^18^. However, little is known about the molecular mechanism of genes involved in lignin biosynthesis in celery leaf development. Celery is a kind of low fat and high fiber food diets. Lignin content in celery has a directly influence on the development of cell wall thickness and xylem, which affect the quality of celery. Better understanding of the lignin biosynthesis mechanisms in celery should help to improve the celery quality.

In this study, celery (‘Ventura') leaves at three stages were collected and analyzed using Illumina paired-end sequencing technology. The leaves were qualitatively evaluated for lignin distribution using histochemistry and autofluorescence microscopy (Figure 1), and then anatomically characterized. We constructed a database of the transcriptome sequences in the ‘Ventura' leaves and then isolated cDNA sequences with highly homologous genes that encode lignin biosynthesis-related enzymes, including PAL, C4H, 4CL, HCT, C3H, CCoAOMT, CCR, CAD, F5H, COMT, and POD. The cDNA sequences were used to analyze the expression profiles of genes involved in lignin biosynthesis in celery by quantitative real-time PCR. Profiling the expression of genes that encode lignin biosynthesis-related enzymes and understanding lignin distribution may help elucidate the regulatory mechanisms of lignin biosynthesis in celery. The results of this study may serve as a guide to develop breeding strategies that improve celery quality.

## Results

### Illumina paired-end sequencing and *de novo* assembly of celery

Transcriptome sequencing was carried out on *A. graveolens* cv ‘Ventura' to reveal the transcriptome differences among the different stages of celery leaves. In this study, 35 512 555, 58 074 820 and 29 938 235 raw reads were obtained from leaves at Stage 1, Stage 2, and Stage 3, respectively. The average length of raw reads was 100 nucleotides. After stringent quality assessment and data filtering, reads with a base quality greater than 20 were selected for further analysis. A total of 32,477,416 quality reads were recorded for the leaves at Stage 1, 53,675,555 at Stage 2, and 27,158,566 at Stage 3, respectively. The quality reads for the leaves at the three stages were combined and used to draw the transcriptome information of ‘Ventura'. The high quality reads were *de novo* assembled by Trinity software, and default setting K-mer value of 25 was used to construct the unique consensus sequences. A K-mer value of 25 improved the N50 and average length by a minimal percentage while avoided the loss in sensitivity. Finally, all short sequences were assembled into 33,213 unigenes with an average length of 1,478 bp, a maximum length of 17,075 bp, and an N50 of 2,060 bp. The length distribution of the unigenes is illustrated in Figure S1. The sequences ranging from 200 bp to 2,700 bp in length accounted for nearly 89.8% of the total. Up to 2,918 unigenes (8.8%) and 466 unigenes (1.4%) were 2,700 bp to 5,000 bp and >5000 bp in length, respectively.

### Functional classification by evolutionary genealogy of genes: Non-Supervised Orthologous Groups (eggNOG), Gene Ontology (GO), Kyoto Encyclopedia of Genes and Genomes (KEGG)

The eggNOG database consists of protein sequences and is encoded in 21 complete genomes, including proteins of bacteria, algae, and eukaryotes. This database was built on classifications according to their evolutionary relationships^19^. To further evaluate the completeness of the transcriptome library and the effectiveness of the annotation process, all annotated unigene sequences were searched against the eggNOG database for functional prediction and classification. A total of 26,804 sequences were assigned to eggNOG classifications (Figure 2). Among the 26 eggNOG categories that were assigned to unigenes, the cluster for function unknown (6,606, 24.65%) was the largest group, followed by general function prediction only (5,123, 19.11%), i.e., basic physiological and metabolic functions; signal transduction mechanisms (2,226, 8.30%); and post-translational modification, protein turnover, and chaperones (1,754, 6.54%). Undetermined (1, 0.01%) represented the smallest group. The unigenes for secondary metabolite biosynthesis, transport, and catabolism accounted for 3.67% (983) of the total functional genes, which are essential for secondary metabolites that contribute to the quality and taste of celery.

GO terms were assigned to assemble unigenes and provided defined ontologies to express gene product properties. GO terms are a dynamically structured control vocabulary that is applied to describe gene product in terms of their associated biological processes, cellular components, and molecular functions^20^. In the present study, 132,740 unigenes with known functions were assigned to one or more ontologies, and each unigene was assigned to a set of GO Slims. The GO enrichment analysis of unigenes is summarized in Figure 3. GO analysis assigned 63,809 unigenes to biological process, 50,684 to cellular component, and 18,247 to molecular function. Metabolic process (18,380 unigenes, 13.85%) and cellular process (16,165 unigenes, 12.18%) were the most highly represented groups under the biological process category. We also identified genes involved in other important biological processes, such as cellular component organization, multicellular organismal development, post-embryonic development, reproduction, response to abiotic stimulus, response to stress, and transport. For the cellular component category, cell and intracellular were the most highly represented groups, followed by cytoplasm and membrane. Regarding molecular function, binding was the most highly represented group, with numerous ligases, hydrolases, oxidoreductases, and transferases annotated. However, a few genes were assigned to the clusters of “abscission”, “fruit ripening”, “extracellular space” and “nuclear envelope” and no genes were found in the clusters of “behavior”, “translation regulator activity”, “proteinaceous extracellular matrix” or “transcription regulator activity” (Figure 3). These GO annotations demonstrate that ‘Ventura' expresses genes that encode diverse structural, regulatory, and stress proteins.

A GO enrichment analysis of the leaves at the three stages is shown in Figure S2. The gene numbers of each subcategory varied, and the structural, regulatory, and stress proteins encoded were diverse among the three stages. Stage 1 and Stage 2 significantly differed in gene number on the clusters of “fruit ripening”, “metabolic process”, “response to biotic stimulus”, “response to external stimulus”, “external encapsulating structure”, “extracellular region”, and “thylakoid”. Meanwhile, Stage 2 and Stage 3 significantly differed in gene number on the clusters of “growth”, “response to endogenous stimulus”, “response to stress”, “sequence-specific DNA binding transcription factor activity”, “external encapsulating structure”, “extracellular region”, and “binding”.

KEGG analysis^21^ demonstrated the biological pathways in which the unigenes are involved. Assembled unigenes were compared with the KEGG database using BLASTx, and the corresponding pathways were established. Among the 10,973 unigenes assigned to KEGG pathways, 3,914 were assigned to metabolism, 2,207 to human diseases, 1,858 to genetic information processing, 1,416 to organismal systems, 962 to cellular processes, and 616 to environmental information processing (Figure 4). Among the pathways that were assigned to the unigenes, carbohydrate metabolism (1,091 unigenes) was the largest group, followed by infectious diseases (976 unigenes), translation (746 unigenes), and energy metabolism (589 unigenes). Signaling molecules and interaction (1) represented the smallest group. These annotations provide a valuable resource for investigating specific processes, structures, functions, and pathways in celery research.

Significant differences in the number of unigenes and their transcript abundances were observed among the three stages (Figure S3). Stage 1 and Stage 2 significantly differed in gene number on the pathways of energy metabolism, biosynthesis of other secondary metabolites, replication and repair, cell growth and death, immune diseases, and substance dependence. Meanwhile, Stage 2 and Stage 3 significantly differed in gene number on the biosynthetic pathways of other secondary metabolites as well as replication and repair. In general, the three stages significantly differed in gene number on the biosynthetic pathways of other secondary metabolites as well as replication and repair. Unigenes functioning in the biosynthesis of other secondary metabolites were further analyzed. The lignin biosynthetic pathway, which is tightly related to celery quality, was selected for further analysis.

### Anatomic characteristics of leaf blades and petioles from ‘Ventura' at three stages

This study comprehensively investigated the structural leaf development of ‘Ventura' using resin-embedding microtomy and scanning electron microscopy (SEM). As shown in Figure 5, the leaf blade gradually thickened, and the spongy and palisade mesophyll tissues were tightly arranged at the three stages. The collenchyma and vascular bundles in the leaf vein thickened and expanded during leaf growth and development. As shown in Figure 6, the collenchyma grew, the vascular bundles further expanded, the phloem and xylem extensively developed, and the epidermal cells of the petioles expanded from Stage 1 to Stage 3.

Petiole transection structure was characterized with SEM (Figure 7).The vascular bundles in petioles serve as important channels for material transport, and collenchyma strengthen and support tissues in plants^22^,^23^. The collenchyma and vascular bundles in the petioles were thick and large, and the cells were large and tightly arranged (Figure 7A–D).

### Lignin distribution in celery leaf identified by histochemistry and autofluorescence microscopy

Lignification has been studied through various microscopic techniques, such as histochemistry, microautoradiography, interference microscopy, UV absorbance, fluorescence microscopy, confocal Raman microscopy, and transmission electron microscopy^24^. In the present study, lignin content was qualitatively evaluated in different tissues during leaf development to investigate the lignin accumulation pattern in celery leaves. Lignified tissues were identified through histochemistry and fluorescence microscopy. Lignin presents a broad fluorescence emission range and is excited with UV. Tissues positive to safranin O-fast green staining as indicated by red stain contained lignin.

The UV-excited fluorescence in the leaf blades and petioles of celery is shown in Figure 8. Autofluorescence was easily observed in the leaves at the three stages. Lignin fluorescence was observed in the vascular bundles and cell walls, and a strong intensity was detected in the xylem. No difference was detected in the location or intensity of lignin autofluorescence in the cell walls of the leaf blades among the three stages (Figure 8A, C, and E). The vascular bundles in the petioles expanded with development and were intensely located in the secondary walls of xylem tissues. The largest density was recorded at Stage 3 (Figure 8B, D, and F). Lignin was deposited in the vascular bundles and the cell walls, and strong lignification was observed in the xylem. No difference in the lignin content in leaf blade cell walls was detected among the three stages. However, the lignin content in petiole vascular bundles increased with development and was particularly high in the secondary walls of xylem tissues. Lignin accumulated in the vascular tissues at Stage 1, and the highest lignin content was recorded at Stage 3.

Safranin O-fast green reagent was used for lignin histochemical staining to qualitatively evaluate lignin content. Leaf blades and petioles were embedded in paraffin, and cross sections were stained with safranin O-fast green. Red staining indicated lignin deposition. As illustrated in Figure 9, the collenchyma and vascular bundles in the petioles showed slight lignification at Stage 1. No significant difference was detected in lignin contents in the collenchymas and epidermis between Stage 1 and Stage 2, but strong lignification was observed at Stage 3 (Figure 9A, C, and E). Lignin accumulation in the vascular bundles increased with the stages, and strong lignification was observed in the xylem (Figure 9B, D, and E). Only a slight deposition in vascular bundle was observed at Stage 1, and considerable deposition was previously observed at Stage 3. As shown in Figure 10, lignin accumulation occurred and gradually increased in the palisade and spongy mesophyll tissues at the three stages. The palisade and spongy tissues showed minimal lignification at Stage 1 but demonstrated strong lignification at Stage 3. No lignin deposition was observed in the leaf vein of the three stages.

Lignin distribution in different tissues and at different leaf developmental stages was qualitatively measured by histochemistry and autofluorescence microscopy. In the leaf blade tissues of celery, lignin accumulation gradually increased in the palisade and spongy tissues at the three stages. However, no difference in the lignin content of cell walls was observed among the three stages. Lignin accumulation increased in the vascular bundles, collenchyma, and epidermis in the petioles at the three stages, and the xylem showed stronger lignification.

### Isolation candidate genes involved in lignin biosynthesis in celery

Monolignol biosynthesis, which contributes to celery taste, was selected for further analysis. The 11 genes involved in lignin biosynthesis are members of multigene families in higher plant (e.g. 9 *CAD* in *Arabidopsis*, 9 *CAD* in rice, 3 *COMT* in oat grass, 5 *PAL* in pine). In celery, the genes (*AgPAL, AgC4H, Ag4CL, AgHCT, AgC3H, AgCCoAOMT, AgCCR, AgCAD, AgF5H, AgCOMT, AgPOD*) involved in lignin biosynthesis maybe also have multiple copies (Figure 11). The *AgPAL, AgC4H, Ag4CL, AgHCT, AgC3H, AgCCoAOMT, AgCCR, AgCAD, AgF5H, AgCOMT, AgPOD* genes were identified using a BLAST-based search tool from the transcriptome data of celery. In this study, the genes involved in lignin biosynthesis in celery showing high homology with the reported genes participated in lignin biosynthesis were selected. These selected genes also showed high RNA transcript levels based on the transcriptome data of different stages of celery.

Our dataset includes annotated sequences for all genes involved in monolignol biosynthesis. These genes displayed high homology to *Arabidopsis* or other dicot genes, and most genes presented more than 80% similarity at the protein level (data not shown). This result suggests that these genes were highly conserved during the evolution. The 11 genes that encode lignin biosynthesis-related enzymes were cloned from ‘Ventura'. The nucleotide sequences of *AgPAL*, *AgC4H*, *Ag4CL*, *AgHCT*, *AgC3H*, *AgCCoAOMT*, *AgCCR*, *AgCAD*, *AgF5H*, *AgCOMT*, and *AgPOD* are listed in Figure S4 to S14.

### Expression profiles of candidate genes involved in lignin biosynthesis in different celery tissues

In the monolignol biosynthetic pathway, quantitative real-time PCR assays were utilized to study the expression profiles of lignin biosynthesis-related genes in different tissues in ‘Ventura'. The following 11 genes involved in lignin biosynthesis were differentially expressed in the roots, stems, petioles, and leaf blades of ‘Ventura': *AgPAL*, *AgC4H*, *Ag4CL*, *AgHCT*, *AgC3H*, *AgCCoAOMT*, *AgCCR*, *AgCAD*, *AgF5H*, *AgCOMT*, and *AgPOD*.

The 11 genes were differentially expressed in the roots, stems, petioles, and leaf blades of ‘Ventura' (Figure 12). These genes may perform different functions in different celery tissues and thus affect the growth and development of celery plants. The relative expression level of *AgCCoAOMT* was higher in the roots than in the other three tissues, and no significant difference in its expression was detected among the stems, petioles, and leaf blades. The transcript levels of *AgCOMT* were the highest in the stems, followed by the petioles and leaf blades, and then the roots. The transcript levels of *AgC4H*, *AgF5H*, and *AgHCT* were higher in the petioles than in the other three tissues. The relative expression levels of *AgC4H* and *AgHCT* were slightly lower in the roots and leaf blades and remained low in the stems. The expression level of *AgF5H* was lower in the roots and stems than in the leaf blades and petioles. The transcript levels of *AgPAL*, *AgC3H*, *Ag4CL*, *AgCCR*, *AgCAD*, and *AgPOD* were higher in the leaf blades than in the other three tissues. The relative expression levels of *AgPAL*, *AgC3H*, *AgCCR*, and *AgCAD* were lower in the roots, stems, and petioles than in the leaf blades, with no significant difference among the three former tissues. The transcript level of *Ag4CL* was slightly low in the stems and remained relatively low in the roots and petioles, with no significant difference between the two latter tissues. The relative expression of *AgPOD* was relatively low in the roots and petioles with no significant difference, and the lowest expression level was observed in the stems (Figure 12).

### Expression profiles of candidate genes involved in lignin biosynthesis at different developmental stages in celery

The 11 genes mentioned above were expressed in the petioles and leaf blades of ‘Ventura' at the three stages.

#### In petiole

The relative transcript level of *AgCOMT* in the petioles increased and peaked at Stage 1, decreased at Stage 2, and then continued to decrease and diminish at Stage 3. The transcript levels of *AgC3H*, *Ag4CL*, and *AgPOD* gradually increased among the three stages, with no significant difference in the relative expression of *AgC3H* and *AgPOD* between the two former stages. The relative expression levels of *AgPAL*, *AgC4H*, *AgF5H*, *AgHCT*, *AgCCoAOMT*, *AgCCR*, and *AgCAD* increased at Stage 2 and then decreased at Stage 3. The transcript levels of *AgPAL*, *AgC4H*, and *AgCCoAOMT* were higher at Stage 3 than at Stage 1, but that of *AgHCT* was lower at Stage 3 than at Stage 1. No significant difference in the transcript levels of *AgF5H*, *AgCCR*, and *AgCAD* was detected between Stage 1 and Stage 3 (Figure 13).

#### In leaf blade

The transcript levels of *AgPAL*, *AgC4H*, and *AgCOMT* in the leaf blades gradually decreased at the three stages. No significant difference in *AgC4H* transcript was found between Stage 2 and Stage 3, and *AgCOMT* transcript was undetected at these two stages. A deficiency in *AgCOMT* reduces the quantity of S monomers and thus affects lignin composition^25^. In other words, the content of S monomers would no longer increase at the two latter stages. The expression levels of *AgF5H* and *Ag4CL* gradually increased at the three stages and peaked at Stage 3. The relative expression levels of *AgC3H* and *AgPOD* decreased at Stage 2 but increased at Stage 3. The transcript levels were slightly lower at Stage 2 than at Stage 1. The transcript levels of *AgHCT*, *AgCCR*, and *AgCAD* increased at Stage 2 and decreased at Stage 3. *AgHCT* transcript level was higher at Stage 1 than at Stage 3. No significant differences in *AgCCR* and *AgCAD* transcript levels was detected at Stage 1 and Stage 3. The relative expression of *AgCCoAOMT* was not significantly different among the three stages (Figure 13).

## Discussion

With the introduction of NGS, transcriptome sequencing has become an important tool in research because of its low cost and high throughput^26^,^27^. Short reads from Illumina high-throughput paired-end sequencing can be well assembled and used for transcriptome analysis and gene identification^28^. In the present study, larger amount raw reads were obtained from celery leaves collected at Stage 1, Stage 2, and Stage 3, respectively. The reads were assembled into 33,213 unigenes with an average length of 1,478 bp, a maximum length of 17,075 bp, and an N50 of 2,060 bp. The *de novo* assembly was proven to be of high quality by comparison with the reported celery databases. The unigenes obtained in the present study were basically in accordance with those reported for celery, but the average length was longer than that reported using the same platform^29^.

Considering the difficulty in estimating the number of genes and predicting the potential functions of the transcripts because of lacking reference genome, we conducted BLAST analysis using the public protein databases to indirectly identify genes. Detailed functional information is important to understand the overall expression profiles of ‘Ventura'^18^,^30^,^31^. Large numbers of unigenes were assigned to various eggNOG^32^, GO^33^, and KEGG^34^ classifications. To evaluate the completeness of the transcriptome library and the effectiveness of the annotation process, annotated unigene sequences were searched against the eggNOG database for functional prediction and classification. A total of 26,804 sequences were assigned to eggNOG classifications^35^. Among the 26 eggNOG categories, the cluster for function unknown represented the largest group. GO terms were assigned to assembled unigenes, and defined ontologies representing gene product properties were provided^36^. In the present study, 132,740 unigenes with known functions were assigned to one or more ontologies, and each unigene was assigned to a set of GO Slims. The gene numbers of each subcategory varied, and the variety of proteins encoded differed among the three stages. KEGG analysis can appropriately demonstrate the various biological pathways^34^. In the present study, 10,973 unigenes were assigned to KEGG pathways. The number of unigenes and their transcript abundances significantly differed among the three stages in ‘Ventura'. The number of genes on the biosynthetic pathways of other secondary metabolites significantly varied among the three stages. Further analysis revealed unigenes involved in lignin biosynthesis^37^.

Lignin accumulation in different tissues and at different stages is closely related to the anatomic characteristics of ‘Ventura'. Lignin is usually distributed in the cell walls of mechanical and vascular tissues. Lignin increases the intensity and the watertightness of cell walls, enhances the mechanical strength of stems, and improves material transport ability^38^. In the present study, lignin distribution in different tissues and at different stages was qualitatively measured by histochemistry and autofluorescence microscopy. In the petioles, the collenchyma increased in size, the vascular bundles further expanded, and the phloem and xylem extensively developed. Consistent with lignin distribution, lignin accumulation increased in the petioles, and the xylem exhibited strong lignification. In leaf blade tissues, the spongy and palisade mesophyll tissues in the leaf blades further developed and increasingly became tightly arranged at the three stages. These results were consistent with that lignin accumulation was gradually increased in the palisade and spongy tissues at the three stages.

Lignin accumulation in different tissues and at different stages is typically correlated with the activity of enzymes involved in lignin biosynthesis. This relationship has been reported in several plants, including *Arabidopsis thaliana*, rice (*Oryza sativa*), poplar (*Populus tomentosa*), jute (*Corchorus capsularis*), and eucalyptus (*Eucalyptus globules*)^39^. In the present study, lignin accumulation positively correlated with the transcription levels of genes that encode lignin biosynthesis-related enzymes in ‘Ventura' (Figure 8–13). Lignin accumulation was gradually increased in the palisade and spongy tissues in the leaf blades at the three stages. Meanwhile, lignin accumulation increased in the vascular bundles, collenchyma, and epidermis, and the xylem exhibited strong lignification. Consistent with the observed lignin accumulation patterns, the expression levels of *AgPAL*, *AgC4H*, *Ag4CL*, *AgHCT*, *AgC3H*, *AgCCoAOMT*, *AgCCR*, *AgCAD*, *AgF5H*, *AgCOMT*, and *AgPOD* remained high in the petioles and leaf blades of ‘Ventura' at the three stages. The transcript levels of these 11 genes were regulated during the leaf stage and remained high prior to the increase in lignin levels, suggesting the important roles of these genes in lignin biosynthesis in celery.

PAL is a rate-limiting enzyme in the phenylpropanoid pathway^40^. In the present study, PAL indirectly participated in monolignol biosynthesis and served as an intermediate link of phenolic biosynthesis in celery. The expression of *AgPAL* was discrepant in the different tissues, and it was higher in the leaf blades than in the other tissues. This finding suggests that this gene has different functions in celery growth and development. 4CL is another key enzyme involved in lignin biosynthesis, the last step of phenylpropanoid metabolism; this enzyme catalyzes 4-cinnamic acid into 4-cinnamoyl CoA and participates in the biosynthesis of other secondary metabolites, such as phytoalexins, flavonoids, and phenylpropanoid^41^. The transcript level of *Ag4CL* gradually increased in the petioles and leaf blades at the three stages; this result coincided with the increasing lignin accumulation at the three stages. Moreover, the transcript level of *Ag4CL* was higher in the leaf blades than in the petioles. *Ag4CL* downregulation can decrease lignin content but increase cellulose content^42^. Therefore, the ratio of lignin to cellulose maybe controlled by regulating *Ag4CL* expression to improve the quality of industrial crops.

CCR and CAD, which specifically catalyze the final steps of monolignol biosynthesis, may determine the total lignin content and composition in a plant. CCR catalyzes the reaction from thioesters, the products of 4CL, to their corresponding cinnamaldehydes^43^. CAD catalyzes the final reduction of hydroxyl-cinnamaldehydes to the corresponding alcohols^37^. In the present study, the transcript levels of *AgCCR* and *AgCAD* were higher in the leaf blades than in the other tissues; in addition, these levels increased at Stage 2 and decreased at Stage 3. Lignin distribution and accumulation were observed in the palisade and spongy tissues of leaves at the three stages but can only be found in the vascular bundles, collenchyma, and epidermis of the petioles. In general, lignin distribution increased in the petioles and leaf blades at the three stages. Repressing the activities of CAD and CCR, which play significant roles in plant lignification, severely affects lignification formation during plant growth and development^44^. The lignin content of celery may be controlled by regulating the transcript levels of *AgCCR* and *AgCAD*, which help improve the comprehensive utilization of celery.

HCT affects lignin content and significantly changes lignin composition. *HCT* downregulation greatly reduces lignin content and markedly increases the proportion of H units as compared with G and S units^45^. *AgHCT* transcripts were higher in the petioles than in the other tissues. The relative expression of *AgHCT* was higher at Stage 2 than at Stage 3, and the transcript level of *AgHCT* was lower at Stage 3 than at Stage 1. The proportion of H units may be higher in the leaf blades than in the petioles, and the increased proportion of H units at Stage 3 may be attributed to *AgHCT* downregulation.

F5H and COMT play key roles in the formation of syringyl units. F5H catalyzes the 5-hydroxylation of coniferaldehyde and coniferyl alcohol, which are then methylated by COMT^25^. The reactions either directly generate sinapyl alcohol or produce sinapaldehyde, which is reduced to sinapyl alcohol by the action of CAD^46^. *AgF5H* or *AgCOMT* downregulation strongly reduces S-unit content, whereas F5H upregulation increases S-unit content^47^. The transcript level of *AgF5H* gradually increased at the three stages and peaked at Stage 3. The transcript level of *AgCOMT* was undetected in the petioles at Stage 3 and in the leaf blades at Stage 2 and Stage 3. In celery, G units but not S units would be produced in the petioles at Stage 3 and in the leaf blades at Stage 2 and Stage 3.The lignin composition in the different tissues maybe controlled by *AgF5H* or *AgCOMT* regulation.

Lignin biosynthesis is closely related to the quality of celery, a vegetable rich in various nutrients, such as vitamin C, folic acid, beta-carotene, calcium, magnesium, and potassium^48^. The taste of celery is affected by cellulose and lignin contents, whereas the quality of this vegetable is considerably influenced by lignin synthesis and related enzyme activity^49^. The main eaten part of celery were leaves, such as petioles and leaf blades. During celery growth and development, lignin content of the cell walls increased and led increased cell walls thickness. Then, tissues of celery become lignified with the increasing lignin concentration. At sufficient lignin concentration, celery leaves become lignified and inedible. Regulating lignin synthesis in celery growth and development is important to the quality of celery.

Celery is an important vegetable with low calorie and rich in dietary fiber, which will help human to keep healthy. In China, ‘Ventura', ‘Liuhe Huangxinqin' and ‘Jinnan Shiqin' were the important three celery cultivars. ‘Liuhe Huangxinqin' and ‘Jinnan Shiqin' were originated from China, while ‘Ventura' is originated from the United States and introduced to China. ‘Ventura' and ‘Jinnan Shiqin' have similar phenotypes. ‘Liuhe Huangxinqin' is a local cultivar from Nanjing, which distinguishes it from the other two celeries in phenotypes^18^,^30^. Till now, ‘Ventura' is well known for the main celery line used to genetic research^30^. Here, to investigate the lignin biosynthesis during celery leaf development, ‘Ventura' was selected for *de novo* assembly, transcriptome characterization, lignin accumulation, and anatomic characteristics.

In this research, we elucidated the mechanism of lignin formation at the different growth and development stages of celery. The corresponding enzymes and genes involved in lignin biosynthesis were also identified. The lignin biosynthesis in celery was regulated by the genes that encode the key enzymes involved in this process (Figure 11). The monolignols are synthesized from phenylalanine through corresponding enzymes of hydroxylation (C4H, C3H, F5H, 4CL, and HCT), O-methylation (COMT and CCoAOMT) and reduction reactions (CCR and CAD), and finally lignin is formed by the dehydrogenation polymerization enzymes (POD or LAC)^12^,^13^,^16^,^17^. Profiling the expression of genes that encode lignin biosynthesis-related enzymes and understanding lignin distribution may help elucidate the regulatory mechanisms of lignin biosynthesis in celery. Our study also supplied useful resources for understanding the mechanism of lignin biosynthesis and the interaction of these genes involved in plants.

## Methods

### Plant material preparation

Seeds of ‘Ventura' were deposited in the State Key Laboratory of Crop Genetics and Germplasm Enhancement, Nanjing Agricultural University, Nanjing, China. The seeds were sown in plastic basins containing peat:vermiculite (2:1,v/v) and then grown in an artificial climate chamber in April 2014. The artificial climate chamber was programmed for 16 h/8 h at 25°C/15°C for day/night and 3000 lux of light intensity. Management of fertilizer and water conditions was consistent.

**Figure 1:**
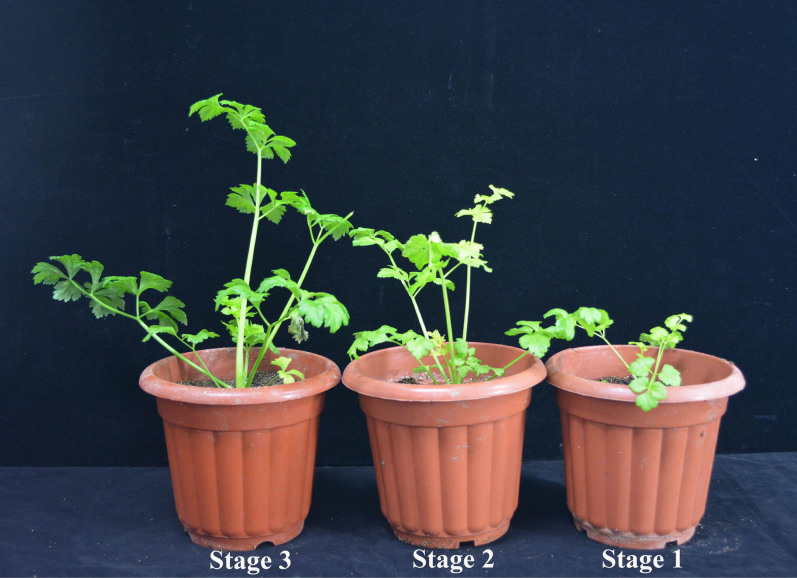
‘Ventura' at three stages.

**Figure 2:**
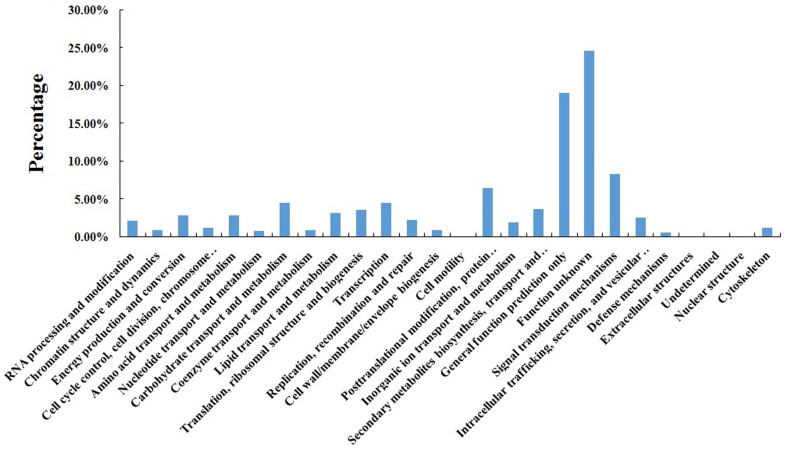
EggNOG classification assigned to unigenes.

**Figure 3:**
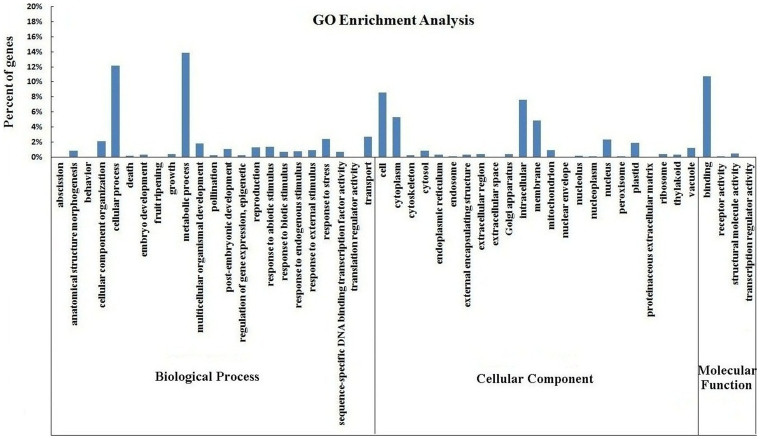
Gene ontology classification of assembled unigenes. Unigenes were summarized into three main categories (biological processes, cellular components, and molecular function) and 50 subcategories. The *x*-axis represents the unigenes' respective categories, whereas the *y*-axis denotes the percentage of unigenes.

**Figure 4:**
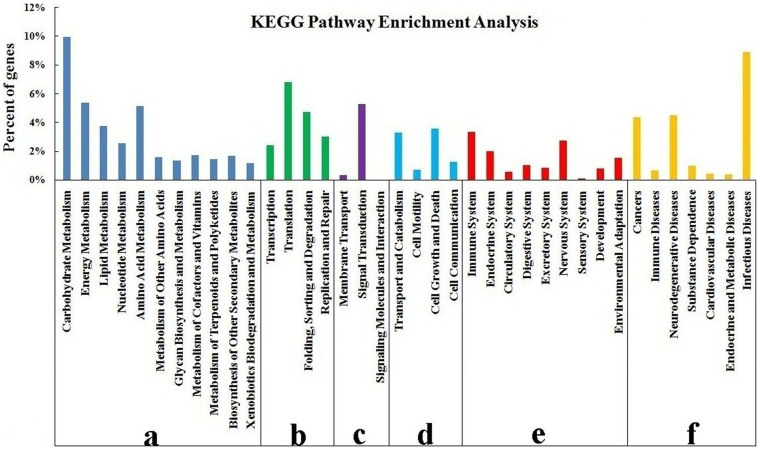
KEGG classification of assembled unigenes. The unigenes were summarized into six main categories (a: metabolism, b: genetic information processing, c: environmental information processing, d: cellular processes, e: organismal systems, and f: human diseases). The *x*-axis represents the unigenes' respective categories, whereas the *y*-axis represents the percent of unigenes.

**Figure 5:**
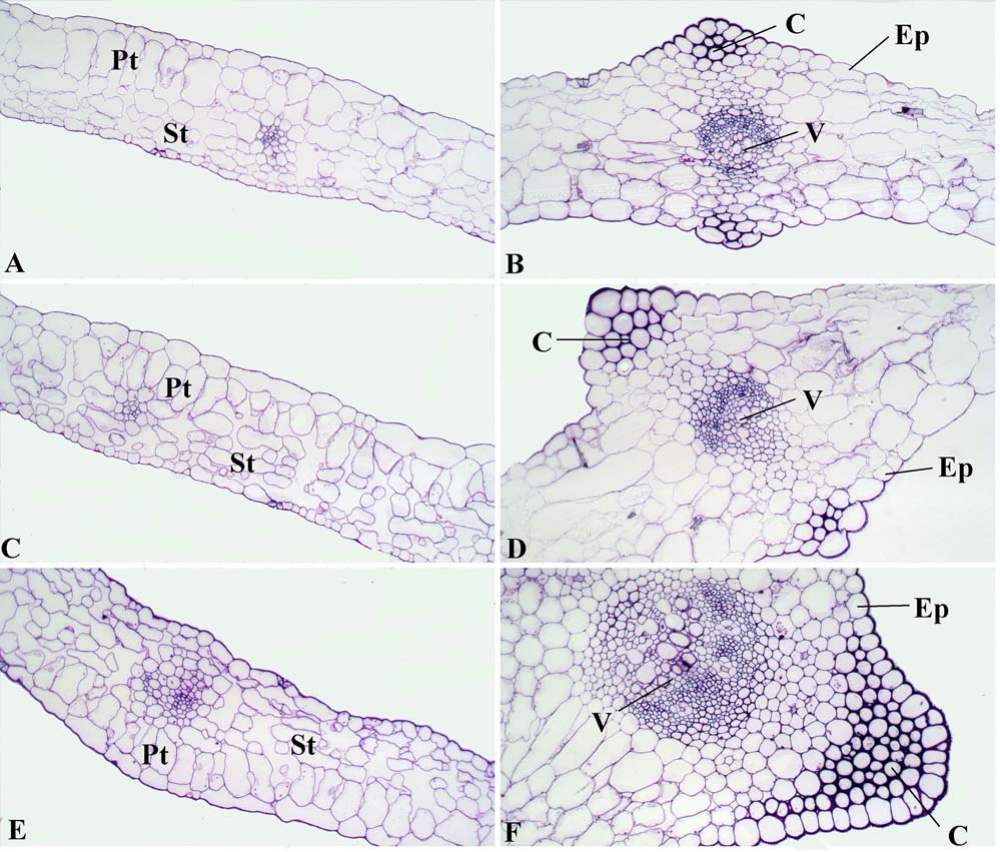
Structural comparison of ‘Ventura' leaf blade. (A): Mesophyll of Stage 1 × 200; (B): Leaf vein of Stage 1 × 200; (C): Mesophyll of Stage 2 × 200; (D): Leaf vein of Stage 2 × 200; (E): Mesophyll of Stage 3 × 200; (F): Leaf vein of Stage 3 × 200. Pt: palisade tissue; St: spongy tissue; Ep: epidermis; V: vascular bundles; C: collenchyma.

**Figure 6:**
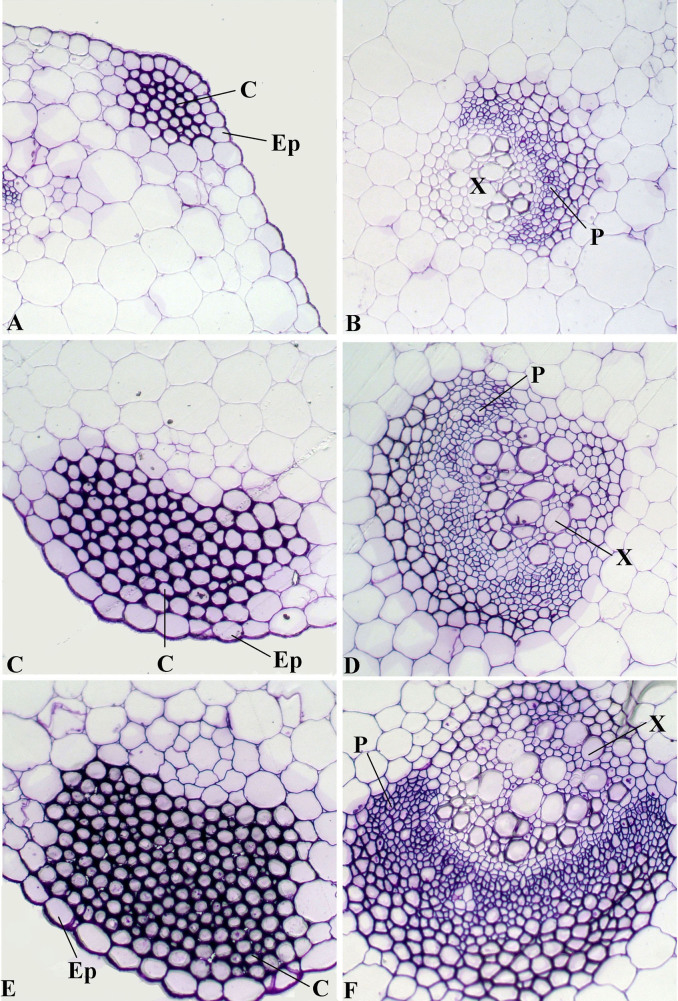
Structural comparison of ‘Ventura' petiole. (A), (B): Stage 1 of ‘Ventura' × 200; (C), (D): Stage 2 of ‘Ventura' × 200; (E), (F): Stage 3 of ‘Ventura' × 200. Ep: epidermis; C: collenchyma; P: phloem; X: xylem.

**Figure 7:**
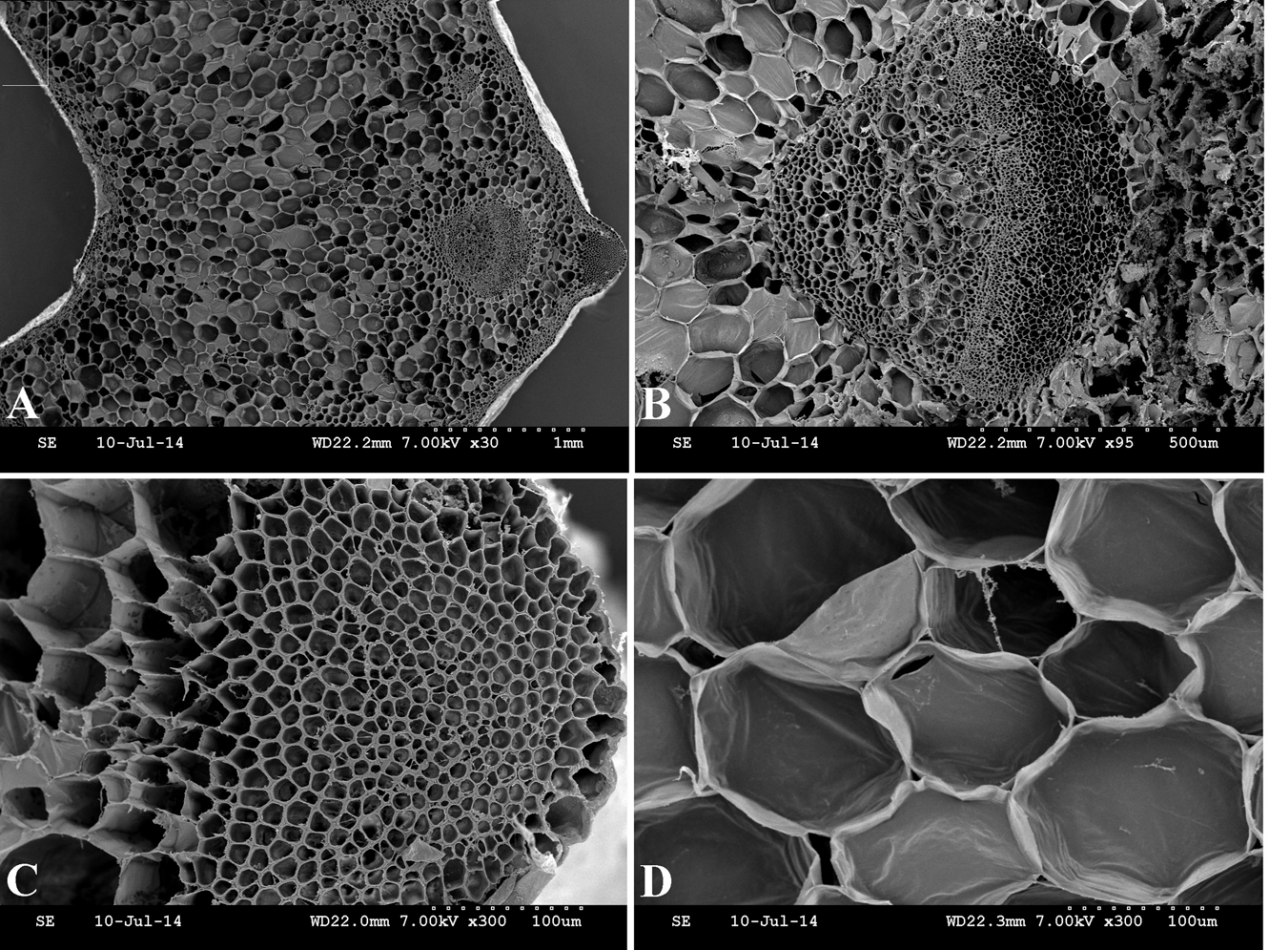
Scanning electron micrographs of ‘Ventura' petiole at Stage 3. (A): Structure of petiole transverse sections; (B): structure of vascular bundle; (C): structure of collenchyma; (D): structure of cells.

**Figure 8:**
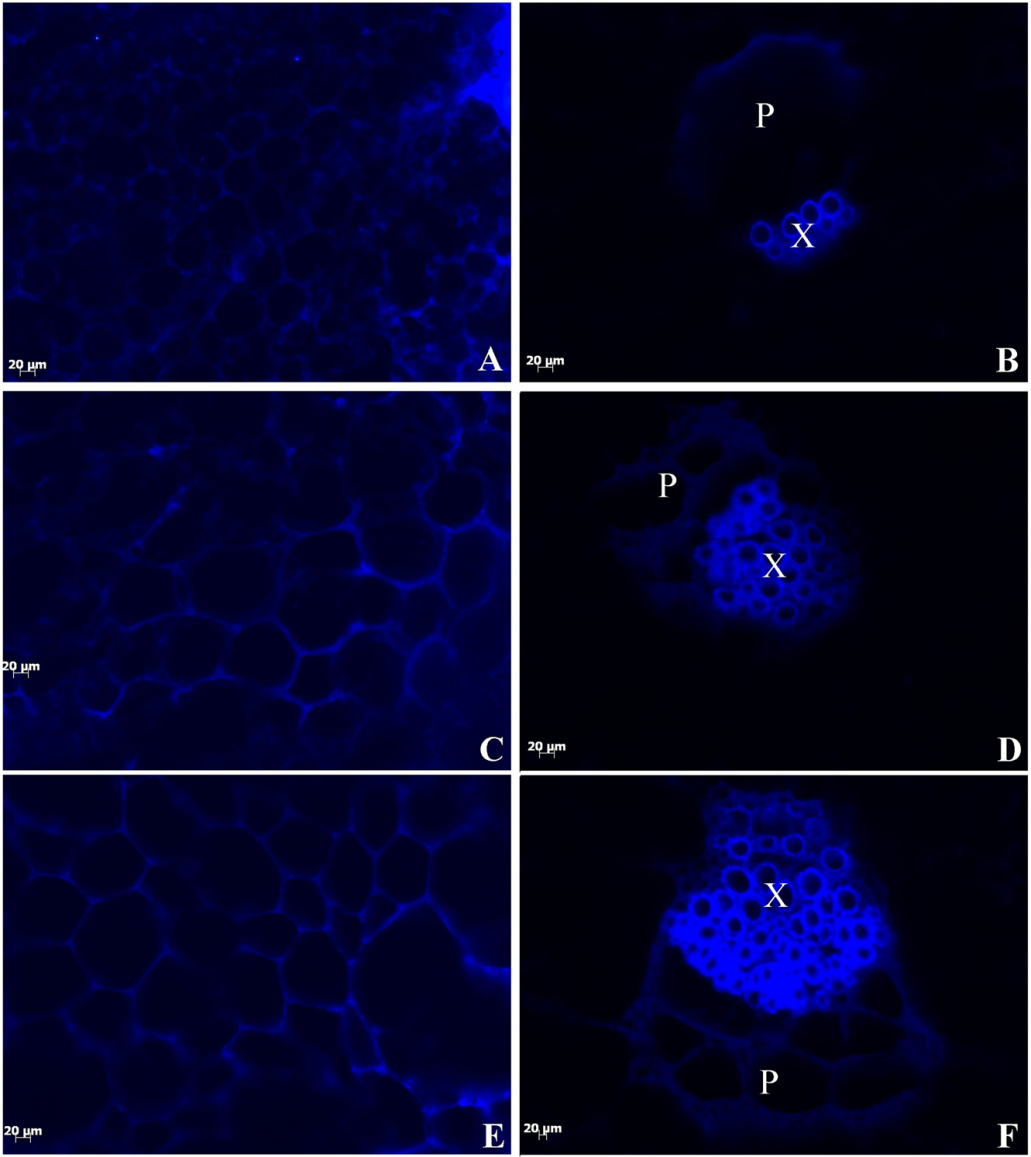
Fluorescence micrographs showing surface and transverse sections of ‘Ventura'. Lignin autofluorescence was visualized following ultraviolet excitation at 365 nm (Scale bar = 20 μm). (A), (C), (E): Fluorescence micrographs showing surface sections of leaf blade; (B), (D), (F): Fluorescence micrographs showing transverse sections of petiole. (A), (B): Stage 1 of ‘Ventura'; (C), (D): Stage 2 of ‘Ventura'; (E), (F): Stage 3 of ‘Ventura'. P: phloem; X: xylem.

**Figure 9:**
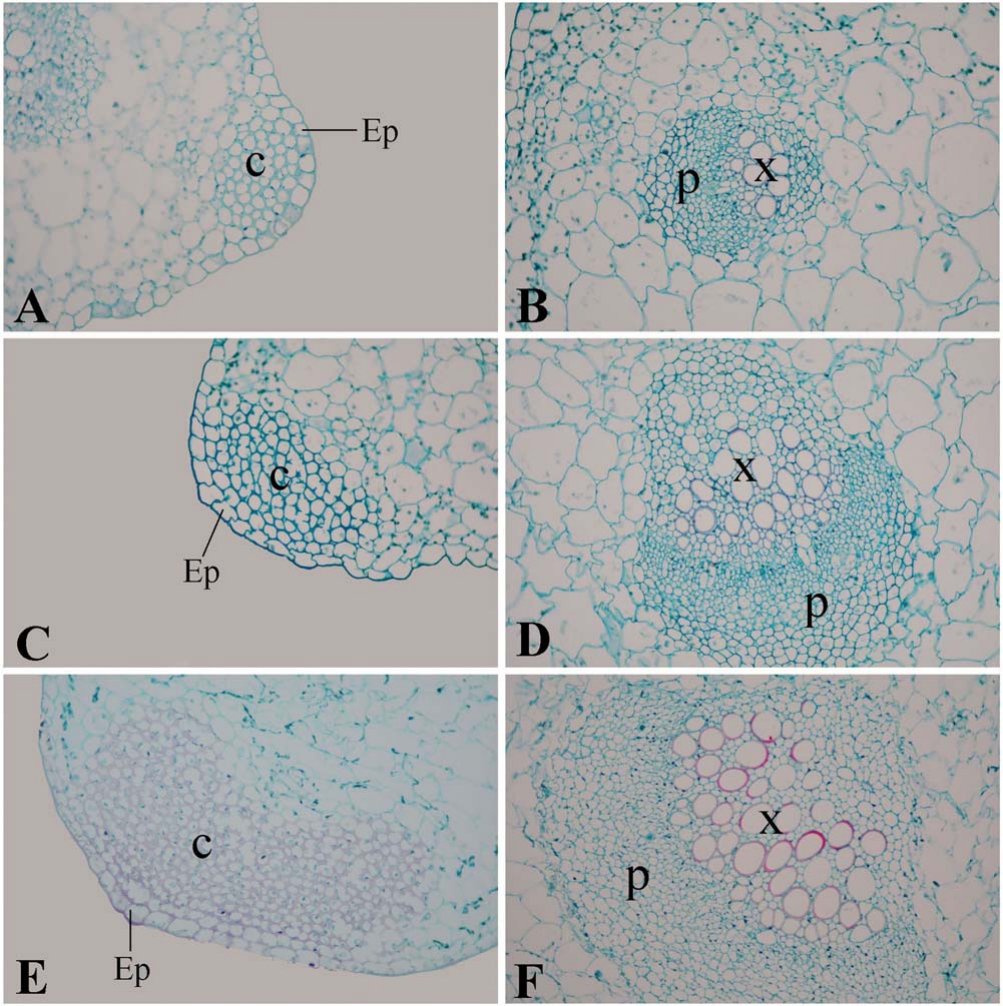
Safranin O-fast green staining of lignin in petiole cross section at three stages of ‘Ventura'. (A), (B): Stage 1 of ‘Ventura' × 40; (C), (D): Stage 2 of ‘Ventura' × 40; (E), (F): Stage 3 of ‘Ventura' × 40. C: collenchyma; Ep: epidermis; P: phloem; X: xylem.

**Figure 10:**
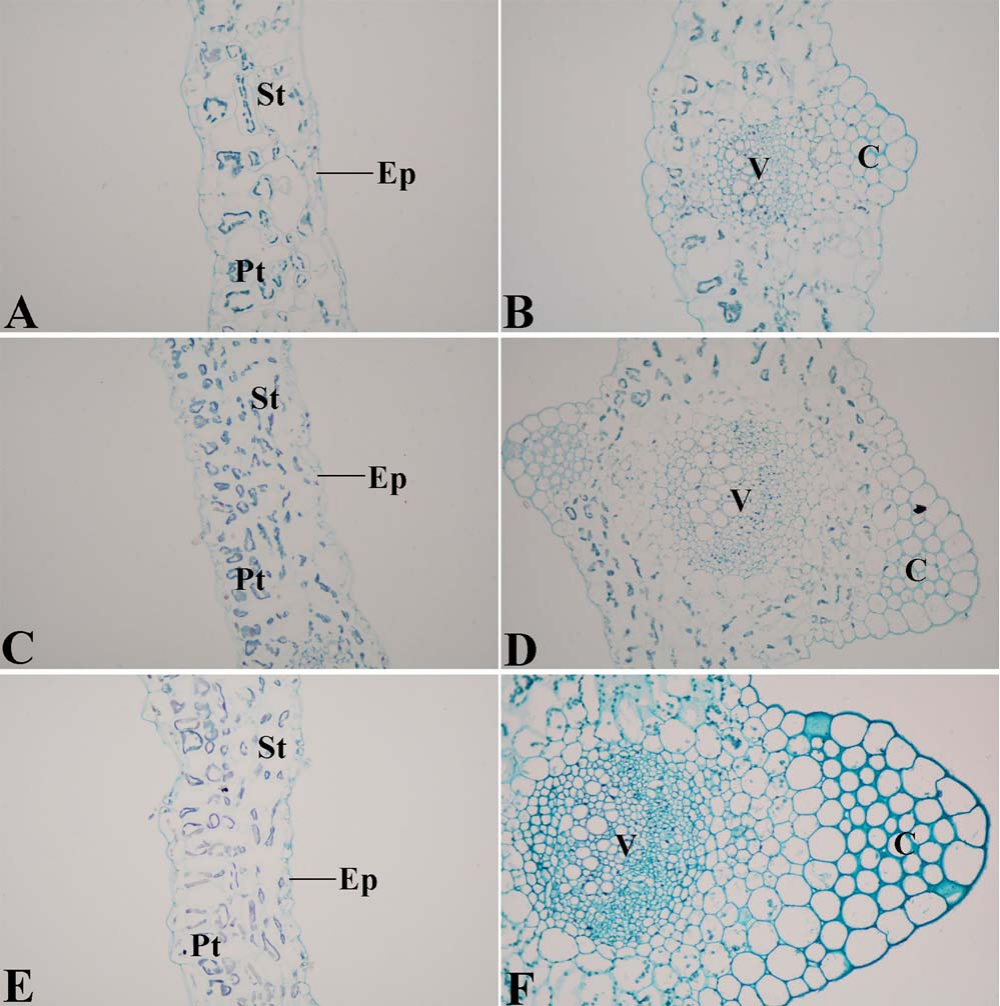
Safranin O-fast green staining of lignin in leaf blade cross section at three stages of ‘Ventura'. (A): mesophyll of Stage 1 × 40; (B): leaf vein of Stage 1 × 40; (C): mesophyll of Stage 2 × 40; (D): leaf vein of Stage 2 × 40; (E): mesophyll of Stage 3 × 40; (F): leaf vein of Stage 3 × 40. Ep: epidermis; Pt: palisade tissue; St: spongy tissue; C: collenchyma; V: vascular bundles.

In this study, the leaf developmental stages of celery were defined as follows: Stage 1: leaf length was 10 cm, Stage 2: leaf length was 20 cm; and Stage 3: leaf length was 30 cm (Figure 1). Leaves at the three stages were selected for transcriptome analysis and sequenced with an Illumina Hi-seq 2000 platform (Shanghai Personal Biotechnology Co., Ltd.). The petioles and leaf blades at each stage and in different tissues (root, stems, petioles, and leaf blades) in ‘Ventura' were obtained, immediately frozen in liquid nitrogen, and then stored at −80°C until use for total RNA isolation. Petioles and leaf blades at the three stages were prepared for resin-embedded sections stained with methylviolet, paraffin sections stained with safranin O-fast green, scanning electron micrographs, and fluorescence micrographs.

### Transcriptome sequence processing and annotation

#### Illumina reads processing and assembly

A Perl script was written to remove low-quality sequences (reads with a base quality less than 20), and all sequences ≤ 50 bp in length were discarded. Quality reads were assembled into contigs, transcripts, and unigenes with Trinity software (http://trinityrnaseq.sf.net). Multiple k-mers potentially guarantee high sensitivity, particularly against low-expressed genes^50^. *De novo* assembly was carried out using a k-mer value of 25 RPKM (reads per kilobase of exon model per million mapped reads) to normalize the abundance of transcripts^51^. A two fold differential was used to identify differentially expressed genes between two stages.

#### Functional annotation and classification

Functional annotations were conducted by comparing sequences with public databases. All Illumina-assembled unigenes were compared with the NCBI nonredundant protein database (http://www.ncbi.nlm.nih.gov/), KEGG database (http://www.genome.jp/kegg), and eggNOG database(http://eggnog.embl.de/) using NCBI Blast(http://www.ncbi.nlm.nih.gov/)^52^.

The orthologous gene products are classified in the eggNOG database^32^. The unigenes were aligned to the eggNOG database to predict and classify the possible functions of the unigenes (http://eggnog.embl.de/version-3.0/, http://www.ncbi.nlm.nih.gov/COG/).

GO offers a dynamic-updated controlled vocabulary and comprehensively describes the properties of genes and their products in any organism. Unigenes can be classified according to GO terms within molecular functions, cellular components, and biological processes^53^. Functional category assignment was conducted using Blast2GO (http://www.blast2go.com/).

The KEGG database contains metabolic pathways, which represent molecular interactions and reaction network. Pathway assignments were carried out on the basis of the KEGG database (http://www.genome.jp/kegg). Unigenes were assigned to special biochemical pathways according to the KEGG database using BLASTx, and then a Perl script, retrieving KO (KEGG Orthology) information from BLASTx results, was developed. We established the pathway correlation between unigenes and database^54^.

### Isolated the genes involved in lignin biosynthesis from ‘Ventura'

**Figure 11:**
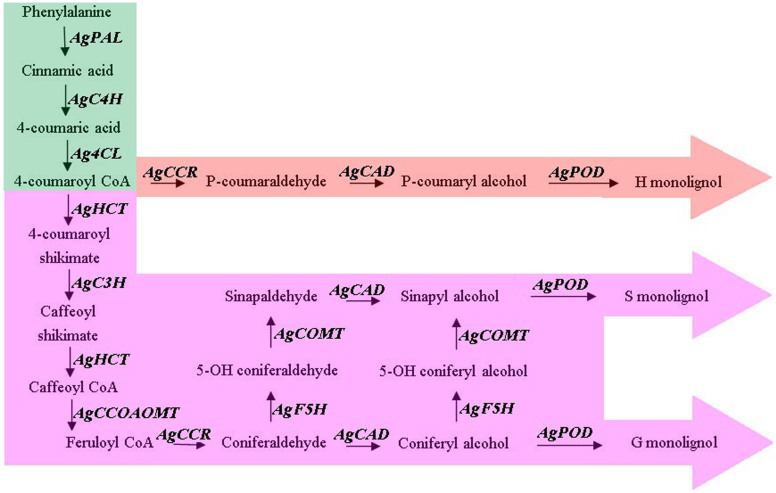
Monolignol biosynthetic pathway in celery. Genes involved in this pathway include *AgPAL*, *AgC4H*, *Ag4CL*, *AgHCT*, *AgC3H*, *AgCCoAOMT*, *AgCCR*, *AgCAD*, *AgF5H*, *AgCOMT*, *AgPOD*, which respectively encode PAL, C4H, 4CL, HCT, C3H, CCoAOMT, CCR, CAD, F5H, COMT, and POD. Abbreviations: PAL, phenylalanine ammonia lyase; C4H, cinnamate4-hydroxylase; 4CL, 4-coumarate:coenzyme A ligase; HCT, hydroxycinnamoyl CoA shikimate/quinate hydroxycinnamoyl transferase; C3H, P-coumarate 3-hydroxylase; CCoAOMT, caffeoyl-CoA O-methyltransferase; CCR, cinnamoyl-CoA reductase; CAD, cinnamyl alcohol dehydrogenase; F5H, ferulate5-hydroxylase; COMT, caffeicacid 3-O-methyltransferase; POD, peroxidase.

**Figure 12:**
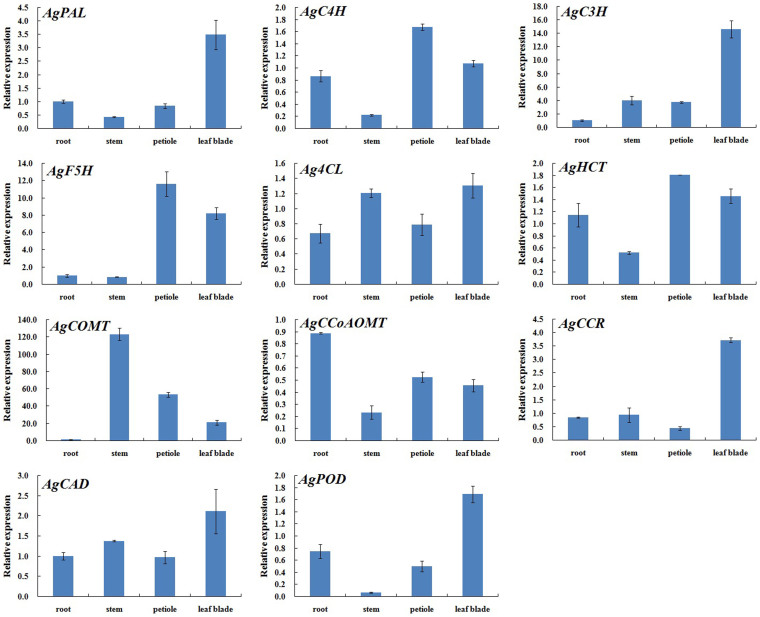
Expression profiles of genes involved in lignin biosynthesis in different tissues of ‘Ventura'. Each bar represents the mean value results from triplicate experiments ± SD.

**Figure 13:**
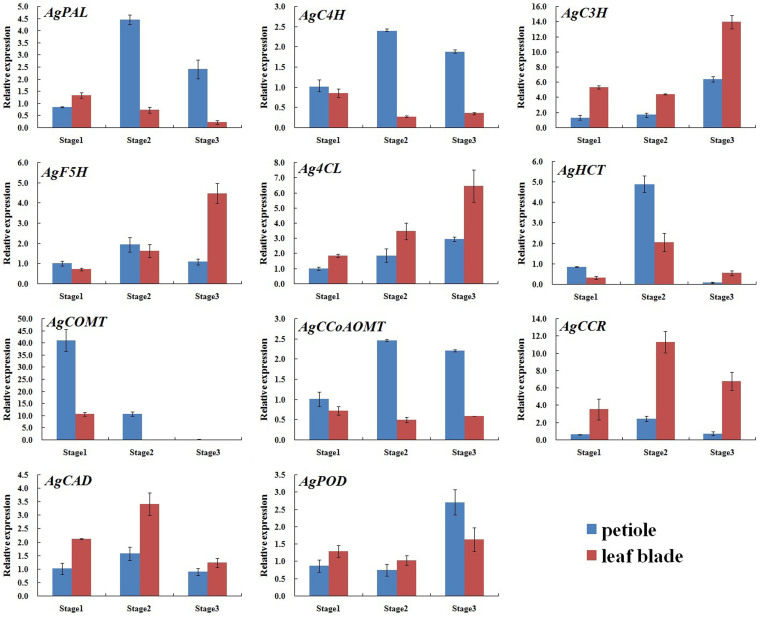
Expression profiles of genes involved in lignin biosynthesis at different stages of ‘Ventura'. Each bar represents the mean value results from triplicate experiments ± SD.

Genes involved in monolignol biosynthesis and celery quality were analyzed as illustrated in Figure 11. *Arabidopsis* or other dicot protein sequences from public databases were aligned with their corresponding genes, which were obtained from key word searches using TBLASTN (E-value threshold of 1e^−5^)^55^,^56^. Genes that encode lignin biosynthesis-related enzymes were cloned from ‘Ventura'. Primers were designed by Primer Premier 5.0 (Table 1).

cDNA sequences of ‘Ventura' were used as templates for PCR amplification. The reaction volume for PCR amplification was 50 μL:25 μL of Premix *Ex Taq*, 2 μL of template cDNA, 1 μL of each primer (20 μM), and 21 μL of sterile distilled water. The PCR reactions were run using the following program: denaturation at 94°C for 5 min; 35 cycles of 30 s at 94°C, 30 s at *Tm* (annealing temperature), and 90 s at 72°C; and a final step at 72°C for 10 min. The PCR products were detected and recovered by 1.2% agarose gel electrophoresis, ligated into a pMD19-T vector, and then transformed into *Escherichia coli*. After extracting the plasmid, PCR detection was conducted, and the bacteria liquid was sequenced. Primer synthesis and gene sequencing were accomplished by GenScript (Nanjing, China).

### RNA extraction and quantitative real-time PCR analysis

The total RNA sequences were extracted by an RNAsimple Total RNA Kit (Tiangen-bio, Beijing, China) according to the manufacturer's instructions. RNA quality and purity were assessed with OD260/280 ratio and determined using a Nanodrop 2000 spectrophotometer (Thermo Scientific, Wilmington, DE). Approximately 10 μg of each sample was reverse transcribed into cDNA using a Prime Script RT reagent Kit (TaKaRa, Dalian, China).

The genes that encode lignin biosynthesis-related enzymes were subjected to quantitative real-time PCR analysis to confirm their expression. Quantitative real-time PCR was experimented to analyze the gene expression at the transcript level in an ABI 7300 Real-Time PCR System and 7300 System software. It was conducted in accordance with the manufacturer's instructions of a Real-Time PCR Kit (SYBR Green) (TaKaRa, Dalian, China). Primers were designed by Primer Premier 5.0 according to the cloned sequences derived from the genes that encode lignin biosynthesis-related enzymes in celery and selected after testing their specificity at 58°C. *Agactin* was used as an internal control. The primers used for quantitative real-time PCR are listed in Table 2. PCR reactions were performed as follows: 94°C for 30 s, followed by 40 cycles of 94°C for 5 s, and 58°C for 30 s. A melting curve (61cycles at 65°C for 10 s) was generated to check amplification specificity. Each reaction had three biological repeats. *Ct* values were represented by the mean values of three independent replicates, and the relative gene expression levels were calculated using the _ΔΔ_Ct method^57^. The standard errors of the mean among the replicates were calculated. Primers were synthesized by GenScript (Nanjing, China).

### Resin-embedding microtomy

In this study, resin-embedding sections were prepared to analyze the inner structure of celery. Petioles and leaf blade specimens at the three stages were cut from healthy ‘Ventura' plants using a razor blade. Petiole specimens were cut from the middle part, leaf blade specimens from the main leaf vein, and mesophyll cells near the vein. Specimens of approximately 2 mm^3^ were prefixed with 2.5% glutaraldehyde at 4°C overnight, which was formulated with 0.05 mol·L^−1^ dimethyl sodium arsenate buffer (pH 7.2). The specimens were washed and dehydrated with an ethanol series of 30% to 100% and then embedded in Spurr's low-viscosity embedding medium^58^. Sections of 1 μm thickness were cut with a glass knife using a Leica Ultracut R (Germany) and then stained with 0.5% methylviolet for 10 min. The structures of the petioles and leaf blades were observed from the sections, and pictures were taken using a CCD camera under a Leica DMLS light microscope.

### Scanning electron microscopy

Petiole transection structures were observed through SEM. Petiole specimens at Stage 3 were vacuum fixed in 3% glutaraldehyde at 4°C overnight and then washed four times with fresh PBS solution. The specimens were postfixed in 1% osmiun tetroxide for 2 h on ice and then washed with fresh PBS solution. The samples were dehydrated in an ethanol series from 30% to 100% and then vacuum dried. The dried samples were installed on aluminum stubs and then coated with gold palladium. Petiole transection structures were examined under a Zeiss DSM-960A SEM at an accelerating voltage of 5 kV. Photographs were collected and assembled using PHOTOSHOP (Adobe, http://www.adobe.com).

### Histochemistry and autofluorescence microscopy

Lignified tissues were identified in the sections through histochemistry and fluorescence microscopy. The petioles and leaf blade specimens at the three stages were fixed in FAA (50 mL of 40% formaldehyde, 50 mL of glacial acetic acid, and 90 mL of 50% ethanol) for 24 h, dehydrated with ethanol, and then embedded in paraffin. Sections of 5 μm thickness were cut with a glass knife using a Leica Ultracut R (Germany).

For safranin O-fast green staining, sections were stained with 1% safranin O in distilled water (pH 6.7) for 10 min, washed, and then counterstained with a 0.1% solution of fast green in water for 5 min. Tissue sections with lignin were stained and observed under a light microscope. The presence of lignin was considered when the tissues were stained with red.

Lignin has a broad range of fluorescence emission and is excited with UV^59^. Xylem-lignified cell walls exhibit autofluorescence^60^. To visualize lignin autofluorescence, tissue sections with lignin were visualized by fluorescence microscopy under UV excitation (330 nm to 380 nm). The fluorescence images were collected with a CCD camera.

## Author Contributions

X.A.S. and X.L.J. initiated and designed the research. X.L.J., G.L.W., F.X., X.R.Y., Z.S.X., F.W. and A.S.X. performed the experiments. X.L.J., W.G.L., F.X. and X.R.Y. analyzed the data. A.S.X. contributed reagents/materials/analysis tools. X.L.J. wrote the paper. X.L.J. and A.S.X. revised the paper.

## Supplementary Material

Supplementary Information Supplementary information

## Figures and Tables

**Table 1:** Primers used for gene cloning

Gene	Direction	Sequence (5′–3′)	Amplicon size (bp)
*AgPAL*	Forward	ATGTTCAGGAACAAGGCTATT	984
	Reverse	TTAGCAGATTGGAAGAGGAGC	
*AgC4H*	Forward	TTGGTGGACTATGCCAAGAAG	1080
	Reverse	CCTAAACTCATTTGGGTTTTT	
*AgC3H*	Forward	TTGGCTAAAGAAGTGTTGAAG	1209
	Reverse	CACCATTCCAGGGTTCTCGCT	
*AgF5H*	Forward	ATGGAAACCAACACTACCGCC	1536
	Reverse	TCAGGAGATAGGACACAACAA	
*Ag4CL*	Forward	ATGGAAACAGTTGACCCGAGA	1635
	Reverse	TCACAGCTTGGAGAGAGATCC	
*AgHCT*	Forward	ATGAAGATCACCGTGAAAGAA	1182
	Reverse	TTAAGCAATTCCTCCAGGTCC	
*AgCOMT*	Forward	ATGGCGCATATCAAGAGTACT	549
	Reverse	CTACTTGCACAATTCCATAAT	
*AgCCoAOMT*	Forward	ATGGCTTCTAATGCTGAATCC	726
	Reverse	TCAGCTGATACGACGGCACAG	
*AgCCR*	Forward	ATGGGTATTGTCCGAACCGAT	1089
	Reverse	CTAAAAGATCTTCTTACAATT	
*AgCAD*	Forward	ATGAGCAAGACGGTGTGCGTA	966
	Reverse	TTAGACAGAAAAAAATTTCTT	
*AgPOD*	Forward	ATGCTGGCTGTGAGTATTACT	963
	Reverse	TTAATTGAAAGCTCTACAAAC	

**Table 2:** Primers used for quantitative real-time PCR analysis

Gene	Direction	Sequence (5′–3′)	Amplicon size (bp)
*Agactin*	Forward	CTTCCTGCCATATATGATTGG	210
	Reverse	GCCAGCACCTCGATCTTCATG	
*AgPAL*	Forward	AATAGACTTGAGGCATTTGGAGG	121
	Reverse	ACAGAATCTTGAGGGATGGAGTT	
*AgC4H*	Forward	TCCTGATTTGGCTAAAGATGT	137
	Reverse	TCTTCCTCCAATGCTCACTAT	
*AgC3H*	Forward	AAGAGGAGCTGGACCGGGTAA	148
	Reverse	TTGGCACTGGCTTTGTGAGGG	
*AgF5H*	Forward	CTTCATTCTTCCGCTGCTTAGTT	136
	Reverse	TTTAGCCAGTCCACGATGAGATA	
*Ag4CL*	Forward	GGCAACTTCTGAAACATTGGTAG	131
	Reverse	GGACCTGGTATCCCTTGTACTTTAT	
*AgHCT*	Forward	TTACTTCCTCCACTCCCACCA	146
	Reverse	TAATCATTGTCCATCCGTGCC	
*AgCOMT*	Forward	GATGAGGGAAAGGTAGTTGTTGC	93
	Reverse	ATAGGCGTCTAACTGAAGGACAG	
*AgCCoAOMT*	Forward	GGCTTCTAATGCTGAATCCAAAC	129
	Reverse	CTAAGCTCTTTCATTGCCTCTGG	
*AgCCR*	Forward	GCTGGGCTTTCTGGCTATTCT	82
	Reverse	TTGCTGCACATGCCTCGATTA	
*AgCAD*	Forward	GGCTACATAGCATCATGGCTTGT	134
	Reverse	TGAAGTCTATCCTTGGCTCCCTC	
*AgPOD*	Forward	TGTTGCTTTGGCGAATGGTCC	98
	Reverse	TAACATCCGGCATATCCTCTG	

## References

[b1] Lattimer J. M. & Haub M. D. Effects of dietary fiber and its components on metabolic health. Nutrients 2, 1266–1289 (2010). 22254008 10.3390/nu2121266 PMC3257631

[b2] Dhingra D., Michael M., Rajput H. & Patil R. T. Dietary fibre in foods: a review. J. Food Sci. Tech. Mys. 49, 255–266 (2012). 10.1007/s13197-011-0365-5 PMC3614039 23729846

[b3] Lin L. Z., Lu S. & Harnly J. M. Detection and quantification of glycosylated flavonoid malonates in celery, Chinese celery, and celery seed by LC-DAD-ESI/MS. J. Agr. Food Chem. 55, 1321–1326 (2007). 17253711 10.1021/jf0624796 PMC3762694

[b4] Dubey D., Shrivastava S., Kapoor S., Dubey P. K. & Jain S. Health promoting properties of common spices. Plant Arch. 7, 13–17 (2007).

[b5] Sowbhagya H. B. Chemistry, technology, and nutraceutical functions of celery (*Apium graveolens* L.): an overview. Crit. Rev. Food Sci. 54, 389–398 (2014). 10.1080/10408398.2011.586740 24188309

[b6] Gase K. & Baldwin I. T. Transformational tools for next-generation plant ecology: manipulation of gene expression for the functional analysis of genes. Plant Ecol. Divers. 5, 485–490 (2012).

[b7] Morozova O. & Marra M. A. Applications of next-generation sequencing technologies in functional genomics. Genomics 92, 255–264 (2008). 18703132 10.1016/j.ygeno.2008.07.001

[b8] Guo S. *et al.* Transcriptome sequencing and comparative analysis of cucumber flowers with different sex types. BMC Genomics 11,384 (2010). 20565788 10.1186/1471-2164-11-384 PMC2897810

[b9] Wang Z., Gerstein M. & Snyder M. RNA-Seq: a revolutionary tool for transcriptomics. Nat. Rev. Genet. 10, 57–63 (2009). 19015660 10.1038/nrg2484 PMC2949280

[b10] Wang Y. M. *et al.* Extensive de novo genomic variation in rice induced by introgression from wild rice (*Zizania latifolia* Griseb.). Genet. 170, 1945–1956 (2005). 10.1534/genetics.105.040964 PMC1449789 15937131

[b11] Yan H. *et al.* Genomic and genetic characterization of rice Cen3 reveals extensive transcription and evolutionary implications of a complex centromere. Plant Cell 18, 2123–2133 (2006). 16877494 10.1105/tpc.106.043794 PMC1560911

[b12] Boudet A. M. Lignins and lignification: Selected issues. Plant Physiol. Bioch. 38, 81–96 (2000).

[b13] Zhao Q. & Dixon R. A. Transcriptional networks for lignin biosynthesis: more complex than we thought? Trends Plant Sci. 16, 227–233 (2011). 21227733 10.1016/j.tplants.2010.12.005

[b14] You T. T., Mao J. Z., Yuan T. Q., Wen J. L. & Xu F. Structural elucidation of the lignins from stems and foliage of *arundo donax* Linn. J. Agr. Food Chem. 61, 5361–5370 (2013). 23646880 10.1021/jf401277v

[b15] Renan Garcia J. *et al.* Rescue of syringyl lignin and sinapate ester biosynthesis in *Arabidopsis thaliana* by a coniferaldehyde 5-hydroxylase from Eucalyptus globulus. Plant Cell Rep. 33, 1263–1274 (2014). 24737414 10.1007/s00299-014-1614-7

[b16] Whetten R. & Sederoff R. Lignin biosynthesis. Plant Cell 7, 1001–1013 (1995). 12242395 10.1105/tpc.7.7.1001 PMC160901

[b17] Boerjan W., Ralph J. & Baucher M. Lignin biosynthesis. Annu. Rev. Plant Biol. 54, 519–546 (2003). 14503002 10.1146/annurev.arplant.54.031902.134938

[b18] Li M. Y. *et al.* High throughput sequencing of two celery varieties small RNAs identifies microRNAs involved in temperature stress response. BMC Genomics 15, 242 (2014). 24673837 10.1186/1471-2164-15-242 PMC3986682

[b19] Venturini L. *et al.* *De novo* transcriptome characterization of *Vitis vinifera* cv. Corvina unveils varietal diversity. BMC Genomics 14, 41 (2013). 23331995 10.1186/1471-2164-14-41 PMC3556335

[b20] Ashburner M. *et al.* Gene ontology: tool for the unification of biology. Nat. Genet. 25, 25–29 (2000). 10802651 10.1038/75556 PMC3037419

[b21] Altman T., Travers M., Kothari A., Caspi R. & Karp P. D. A systematic comparison of the MetaCyc and KEGG pathway databases. BMC Bioinformatics 14, 112 (2013). 23530693 10.1186/1471-2105-14-112 PMC3665663

[b22] Thomas L. H. *et al.* Structure of cellulose microfibrils in primary cell walls from collenchyma. Plant Physiol. 161, 465–476 (2013). 23175754 10.1104/pp.112.206359 PMC3532275

[b23] Zhong R. Q. & Ye Z. H. amphivasal vascular bundle 1, a gain-of-function mutation of the IFL1/REV gene, is associated with alterations in the polarity of leaves, stems and carpels. Plant Cell Physiol 45, 369–385 (2004). 15111711 10.1093/pcp/pch051

[b24] Gierlinger N. & Schwanninger M. Chemical imaging of poplar wood cell walls by confocal Raman microscopy. Plant Physiol 140, 1246–1254 (2006). 16489138 10.1104/pp.105.066993 PMC1435827

[b25] Vanholme R. *et al.* Engineering traditional monolignols out of lignin by concomitant up-regulation of F5H1 and down-regulation of COMT in *Arabidopsis*. Plant J. 64, 885–897 (2010). 20822504 10.1111/j.1365-313X.2010.04353.x

[b26] Galla G. *et al.* Computational annotation of genes differentially expressed along olive fruit development. BMC Plant Biol. 9, 128 (2009). 19852839 10.1186/1471-2229-9-128 PMC2774695

[b27] Kang C. *et al.* Genome-scale transcriptomic insights into early-stage fruit development in woodland strawberry fragaria vesca. Plant Cell 25, 1960–1978 (2013). 23898027 10.1105/tpc.113.111732 PMC3723606

[b28] Liu S., Kuang H. & Lai Z. Transcriptome analysis by illumina high-throughout paired-end sequencing reveals the complexity of differential gene expression during In vitro plantlet growth and flowering in *amaranthus tricolor* L. PLoS One 9, e100919 (2014). 24963660 10.1371/journal.pone.0100919 PMC4071066

[b29] Fu N., Wang Q. & Shen H. L. *De Novo* assembly, gene annotation and marker development using illumina paired-end transcriptome sequences in celery (*Apium graveolens* L.). PLoS One 8, e57686 (2013). 23469050 10.1371/journal.pone.0057686 PMC3585167

[b30] Li M. Y. *et al.* Identification of SSRs and differentially expressed genes in two cultivars of celery (*Apium graveolens* L.) by deep transcriptome sequencing. Hort. Res. 1, 1–9 (2014). 10.1038/hortres.2014.10 PMC4596314 26504532

[b31] Zhuang J. *et al.* Transcriptomic, proteomic, metabolomic and functional genomic approaches for the study of abiotic stress in vegetable crops. Crit. Rev. Plant Sci. 33, 225–237 (2014).

[b32] Jensen L. J. *et al.* eggNOG: automated construction and annotation of orthologous groups of genes. Nucleic Acids Res. 36, D250–D254 (2008). 17942413 10.1093/nar/gkm796 PMC2238944

[b33] Park J. C., Kim T. E. & Park J. Monitoring the evolutionary aspect of the Gene Ontology to enhance predictability and usability. BMC Bioinformatics 9, s7 (2008). 18426552 10.1186/1471-2105-9-S3-S7 PMC2349298

[b34] Wrzodek C., Buechel F., Ruff M., Draeger A. & Zell A. Precise generation of systems biology models from KEGG pathways. BMC Syst. Biol. 7, 15 (2013). 23433509 10.1186/1752-0509-7-15 PMC3623889

[b35] Powell S. *et al.* eggNOG v3.0: orthologous groups covering 1133 organisms at 41 different taxonomic ranges. Nucleic Acids Res. 40, D284–D289 (2012). 22096231 10.1093/nar/gkr1060 PMC3245133

[b36] Harris M. A. *et al.* The Gene Ontology project in 2008. Nucleic Acids Res. 36, D440–D444 (2008). 17984083 10.1093/nar/gkm883 PMC2238979

[b37] Xu Y. *et al.* LtuCAD1 Is a cinnamyl alcohol dehydrogenase ortholog involved in lignin biosynthesis in *liriodendron tulipifera* L., a basal angiosperm timber species. Plant Mol. Biol. Rep. 31, 1089–1099 (2013).

[b38] Xu Z. *et al.* Comparative genome analysis of lignin biosynthesis gene families across the plant kingdom. BMC Bioinformatics 10, s3 (2009). 19811687 10.1186/1471-2105-10-S11-S3 PMC3226193

[b39] Zhao H. Y. *et al.* Isolation and functional characterization of a cinnamate 4-hydroxylase promoter from *Populus tomentosa*. Plant Sci. 168, 1157–1162 (2005).

[b40] Jeong M. J. *et al.* Differential expression of kenaf phenylalanine ammonia-lyase (PAL) ortholog during developmental stages and in response to abiotic stresses. Plant Omics 5, 392–399 (2012).

[b41] Chowdhury M. E. K. *et al.* Regulation of 4CL, encoding 4-coumarate: coenzyme A ligase, expression in kenaf under diverse stress conditions. Plant Omics 6, 254–262 (2013).

[b42] Ambavaram M. M. R., Krishnan A., Trijatmiko K. R. & Pereira A. Coordinated activation of cellulose and repression of lignin biosynthesis pathways in rice. Plant Physiol 155, 916–931 (2011). 21205614 10.1104/pp.110.168641 PMC3032476

[b43] Wang D. D., Bai H., Chen W. Q., Lu H. & Jiang X. N. Identifying a cinnamoyl coenzyme a reductase (CCR) activity with 4-coumaric acid: coenzyme a ligase (4CL) reaction products in *Populus tomentosa*. J. Plant Biol. 52, 482–491 (2009).

[b44] Thevenin J. *et al.* The simultaneous repression of CCR and CAD, two enzymes of the lignin biosynthetic pathway, results in sterility and dwarfism in *Arabidopsis thaliana*. Mol. Plant. 4, 70–82 (2011). 20829305 10.1093/mp/ssq045

[b45] Pu Y., Chen F., Ziebell A., Davison B. H. & Ragauskas A. J. NMR characterization of C3H and HCT down-regulated alfalfa lignin. Bioenerg. Res. 2, 198–208 (2009).

[b46] Franke R. *et al.* Modified lignin in tobacco and poplar plants over-expressing the Arabidopsis gene encoding ferulate 5-hydroxylase. Plant J. 22, 223–234 (2000). 10849340 10.1046/j.1365-313x.2000.00727.x

[b47] Stewart J. J., Akiyama T., Chapple C., Ralph J. & Mansfield S. D. The effects on lignin structure of overexpression of Ferulate 5-Hydroxylase in *hybrid poplar*. Plant Physiol. 150, 621–635 (2009). 19386808 10.1104/pp.109.137059 PMC2689994

[b48] Martelo Castano Y. J., Cortes Rodriguez M. & Suarez Mahecha H. Development of minimally processed celery fortified with vitamine, by matrix engineering. Dyna-Colombia 78, 28–39 (2011).

[b49] Thimm J. C. *et al.* Celery (*Apium graveolens*) parenchyma cell walls: cell walls with minimal xyloglucan. Physiol. Plantarum 116, 164–171 (2002). 10.1034/j.1399-3054.2002.1160205.x 12354192

[b50] Roy R. S., Bhattacharya D. & Schliep A. Turtle: identifying frequent k-mers with cache-efficient algorithms. Bioinformatics 30, 1950–1957 (2014). 24618471 10.1093/bioinformatics/btu132

[b51] Wagner G. P., Kin K. & Lynch V. J. Measurement of mRNA abundance using RNA-seq data: RPKM measure is inconsistent among samples. Theor. Biosci. 131, 281–285 (2012). 10.1007/s12064-012-0162-3 22872506

[b52] Altschul S. F. *et al.* Gapped BLAST and PSI-BLAST: a new generation of protein database search programs. Nucleic Acids Res. 25, 3389–3402 (1997). 9254694 10.1093/nar/25.17.3389 PMC146917

[b53] Conesa A. *et al.* Blast2GO: a universal tool for annotation, visualization and analysis in functional genomics research. Bioinformatics 21, 3674–3676 (2005). 16081474 10.1093/bioinformatics/bti610

[b54] Kanehisa M., Goto S., Furumichi M., Tanabe M. & Hirakawa M. KEGG for representation and analysis of molecular networks involving diseases and drugs. Nucleic Acids Res. 38, D355–D360 (2010). 19880382 10.1093/nar/gkp896 PMC2808910

[b55] Gertz E. M., Yu Y. K., Agarwala R., Schaffer A. A. & Altschul S. F. Composition-based statistics and translated nucleotide searches: improving the TBLASTN module of BLAST. BMC Biology 4, 41 (2006). 17156431 10.1186/1741-7007-4-41 PMC1779365

[b56] Whitney J., Esteban D. J. & Upton C. Recent hits acquired by BLAST (ReHAB): a tool to identify new hits in sequence similarity searches. BMC Bioinformatics 6, 23 (2005). 15701178 10.1186/1471-2105-6-23 PMC549547

[b57] Pfaffl M. W. A new mathematical model for relative quantification in real-time RT-PCR. Nucleic Acids Res. 29, 9 (2001). 10.1093/nar/29.9.e45 PMC55695 11328886

[b58] Spurr A. R. A low-viscosity epoxy resin embedding medium for electron microscopy. J. Ultrastruct. Res. 26, 31–43 (1969). 4887011 10.1016/s0022-5320(69)90033-1

[b59] Donaldson L. A. & Knox J. P. Localization of cell wall polysaccharides in normal and compression wood of radiata pine: relationships with lignification and microfibril orientation. Plant Physiol. 158, 642–653 (2012). 22147521 10.1104/pp.111.184036 PMC3271756

[b60] Saito K. & Fukushima K. Distribution of lignin interunit bonds in the differentiating xylem of compression and normal woods of *Pinus thunbergii*. J. Wood Sci. 51, 246–251 (2005).

