# Supplementary material for: De novo assembly, transcriptome characterization, lignin accumulation, and anatomic characteristics: novel insights into lignin biosynthesis during celery leaf development
Source: Sci Rep. 2015 Feb 5;5:8259. doi: 10.1038/srep08259 (PMC4317703; doi:10.1038/srep08259)
Supplement: Supplementary Information [file srep08259-s1.pdf]

# *De novo* assembly, transcriptome characterization, lignin accumulation, and anatomic characteristics: novel insights into lignin biosynthesis during celery leaf development

Xiao-Ling Jia [1], Guang-Long Wang [1], Fei Xiong [2], Xu-Run Yu [2], Zhi-Sheng Xu [1], Feng Wang [1], Ai-Sheng Xiong*, [1]

1. *State Key Laboratory of Crop Genetics and Germplasm Enhancement, College of Horticulture, Nanjing Agricultural University, Nanjing, 210095, China*
2. *Key Laboratories of Crop Genetics and Physiology of the Jiangsu Province and Plant Functional Genomics of the Ministry of Education, Yangzhou University, Yangzhou 225009, China*

*Please address all correspondence to: A.S. Xiong (xiongaisheng@njau.edu.cn)

-------------

Dr. Ai-Sheng Xiong
Professor
State Key Laboratory of Crop Genetics and Germplasm Enhancement,
College of Horticulture,
Nanjing Agricultural University,
Nanjing, 210095, China
Tel: 86 25 84396790
Fax: 86 25 84396790
Email: xiongaisheng@njau.edu.cn

***Running title:*** *lignin biosynthesis in celery leaf*

# Supplementary Information

**Figure S1: Length distribution of ‘Ventura’ unigenes.**

**Figure S2: Gene ontology classification of assembled unigenes.**

Unigenes were summarized into three main categories (biological processes, cellular components, and molecular function) and 50 subcategories

A: The *x*-axis represents the unigenes’ respective categories, whereas the *y*-axis denotes the percentage of unigenes;

B: The *x*-axis represents the unigenes’ respective categories, whereas and the *y*-axis denotes the number of unigenes.

**Figure S3: KEGG classification of assembled unigenes.**

The unigenes were summarized into six main categories (a: metabolism, b: genetic information processing, c: environmental information processing, d: cellular processes, e: organismal systems, and f: human diseases)

The *x*-axis represents the unigenes’ respective categories, whereas the *y*-axis represents the number of unigenes.

**Figure S4:** Nucleic acid and deduced amino acid sequence of *AgPAL* gene.

**Figure S5:** Nucleic acid and deduced amino acid sequence of *AgC4H* gene.

**Figure S6:** Nucleic acid and deduced amino acid sequence of *AgC3H* gene.

**Figure S7:** Nucleic acid and deduced amino acid sequence of *AgF5H* gene.

**Figure S8:** Nucleic acid and deduced amino acid sequence of *Ag4CL* gene.

**Figure S9**: Nucleic acid and deduced amino acid sequence of *AgHCT* gene.

**Figure S10:** Nucleic acid and deduced amino acid sequence of *AgCOMT* gene.

**Figure S11:** Nucleic acid and deduced amino acid sequence of *AgCCoAOMT* gene.

**Figure S12:** Nucleic acid and deduced amino acid sequence of *AgCCR* gene.

**Figure S13:** Nucleic acid and deduced amino acid sequence of *AgCAD* gene.

**Figure S14:** Nucleic acid and deduced amino acid sequence of *AgPOD* gene.

**Figure S1: Length distribution of 'Ventura' unigenes.**

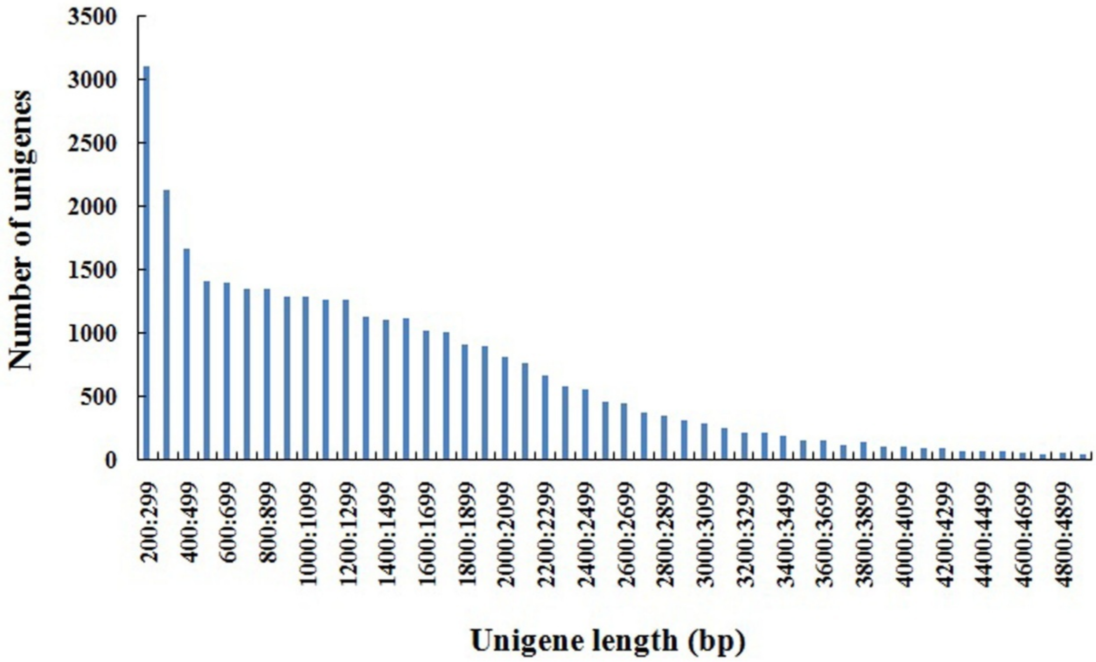

**Figure S2: Gene ontology classification of assembled unigenes.** Unigenes were summarized into three main categories (biological processes, cellular components, and molecular function) and 50 subcategories

A: The *x*-axis represents the unigenes' respective categories, whereas the *y*-axis denotes the percentage of unigenes;

B: The *x*-axis represents the unigenes' respective categories, whereas and the *y*-axis denotes the number of unigenes.

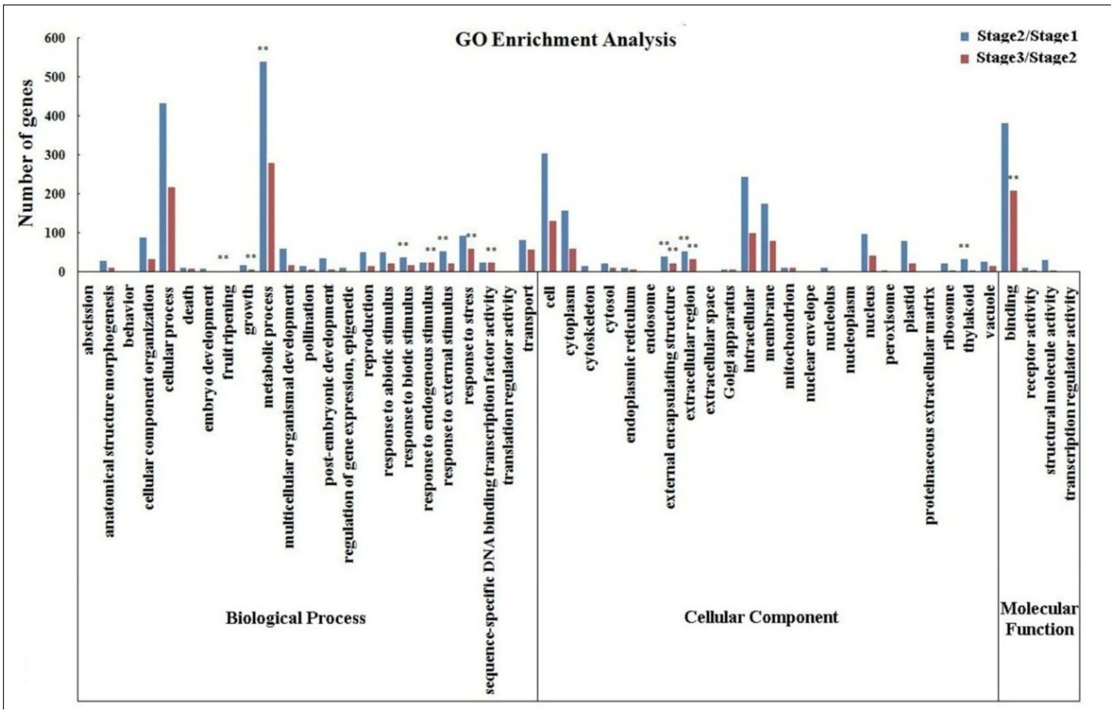

**Figure S3: KEGG classification of assembled unigenes.**

The unigenes were summarized into six main categories (a: metabolism, b: genetic information processing, c: environmental information processing, d: cellular processes, e: organismal systems, and f: human diseases)

The *x*-axis represents the unigenes' respective categories, whereas the *y*-axis represents the number of unigenes.

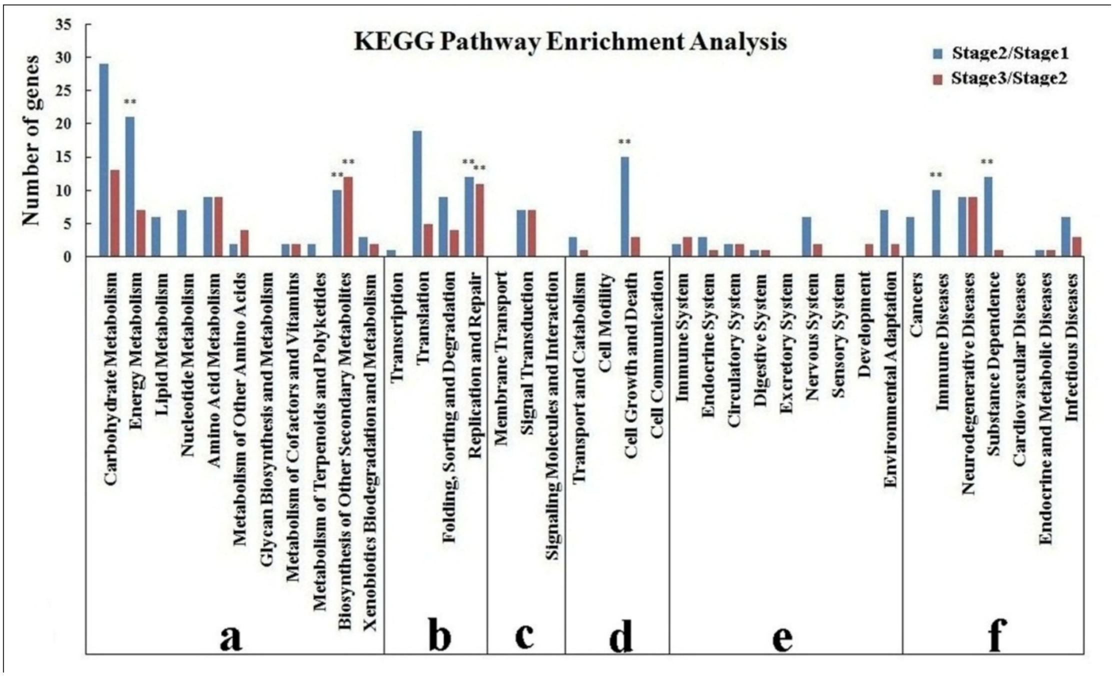

**Figure S4:** Nucleic acid and deduced amino acid sequence of *AgPAL* gene.

```
  1 atgttcaggaacaaggctattcacggtgggaatttcagggcacac
    M  F  R  N  K  A  I  H  G  G  N  F  R  A  H
 46 tattggagtgtcgatggacaatacacgtttggctatgcagcgata
    Y  W  S  V  D  G  Q  Y  T  F  G  Y  A  A  I
 91 ggaaagctcatgtttgctcaattttctgaacttgtaaacgatttt
    G  K  L  M  F  A  Q  F  S  E  L  V  N  D  F
136 tacaacaacgggttgccatcaaacttgtcaggagggcgtaatcca
    Y  N  N  G  L  P  S  N  L  S  G  G  R  N  P
181 agtttggattatggattcaagggtgctgaaattgcgatggcttct
    S  L  D  Y  G  F  K  G  A  E  I  A  M  A  S
226 tactgctctgaacttcaatttttagccaatccagtcaccaaccat
    Y  C  S  E  L  Q  F  L  A  N  P  V  T  N  H
271 gttcaaagtgctgaacagcacaatcaagatgtgaactccttaggc
    V  Q  S  A  E  Q  H  N  Q  D  V  N  S  L  G
316 ttaatctcctcaaggaaaacatcagaagctgttgaaatcttgaaa
    L  I  S  S  R  K  T  S  E  A  V  E  I  L  K
361 ctcatgtctacgacatttttagtaggcctctgccaatcaatagac
    L  M  S  T  T  F  L  V  G  L  C  Q  S  I  D
406 ttgaggcatttggaggagaatttaagaagcactgtaaaaaacaca
    L  R  H  L  E  E  N  L  R  S  T  V  K  N  T
451 gtaagccaagtagctaagcgagtactcaccatgggtgtcaacggg
    V  S  Q  V  A  K  R  V  L  T  M  G  V  N  G
496 gaactccatccctcaagattctgtgagaaagatttactcagagtt
    E  L  H  P  S  R  F  C  E  K  D  L  L  R  V
541 gtggaccgcgaatacatttttgcatatattgatgatccctgcagc
    V  D  R  E  Y  I  F  A  Y  I  D  D  P  C  S
586 gcaacctacccattgatgcaaaaactaaggcaaactctagttgag
    A  T  Y  P  L  M  Q  K  L  R  Q  T  L  V  E
631 catgctttgaagaatggcgacaatgagagaaacttaggcacttcc
    H  A  L  K  N  G  D  N  E  R  N  L  G  T  S
676 atcttccaaaagatagcgacctttgaggatgaactaaaagccata
    I  F  Q  K  I  A  T  F  E  D  E  L  K  A  I
721 ttgcctaaagaagttgaaagtgccagagctgcagtcgaaagtgga
    L  P  K  E  V  E  S  A  R  A  A  V  E  S  G
766 aatccagcaatcccaaacaggatcgaggaatgcaggtcttaccca
    N  P  A  I  P  N  R  I  E  E  C  R  S  Y  P
811 ttgtacaagtttgtcaggaaagagctgggaactgaatatcttaca
    L  Y  K  F  V  R  K  E  L  G  T  E  Y  L  T
856 ggagaaaaagtaacgtcgcctggagaagagttcgataaggtgttt
    G  E  K  V  T  S  P  G  E  E  F  D  K  V  F
```

901 acagccatgagcaaaggagagatcattgatccattgttagagtgc
T A M S K G E I I D P L L E C
946 ctcgagtcatggaatggcgctcctcttccaatctgctaa 984
L E S W N G A P L P I C *

**Figure S5:** Nucleic acid and deduced amino acid sequence of *AgC4H* gene.

1 ttggtggactatgccaagaagtttggagacttgtttatgttacgt
L V D Y A K K F G D L F M L R
46 atgggtcagaggaacttggttgttgtgtcctctcctgatttggct
M G Q R N L V V V S S P D L A
91 aaagatgttttgcatactcagggtgttgagtttggatcacgcacc
K D V L H T Q G V E F G S R T
136 cgtaacgttgttttcgatatcttcacaggtaagggacaggacatg
R N V V F D I F T G K G Q D M
181 gtgttcacagtctatagtgagcattggaggaagatgaggagaatc
V F T V Y S E H W R K M R R I
226 atgacagtacctttctttactaacaaagttgtgcagcagtatcga
M T V P F F T N K V V Q Q Y R
271 tttgggtgggaggatgaggctgctcgtgttgtcgaggatgttaag
F G W E D E A A R V V E D V K
316 gccaatcctgaggctgctacaaaagggatcgtgttgaggaaccgg
A N P E A A T K G I V L R N R
361 ttgcagttgctcatgtataataatatgtacagaatcatgtttgat
L Q L L M Y N N M Y R I M F D
406 agaaggtttgagagtgtagatgatcctttgttcttgaagcttaag
R R F E S V D D P L F L K L K
451 gctttgaatggggagcgcagtaggcttgctcagagcttcgagtat
A L N G E R S R L A Q S F E Y
496 aatttcggagattttatccctattcttcgccctttcttgaggggt
N F G D F I P I L R P F L R G
541 tatcttaaactttgccaggagatcaaggacaaaaggttgaagctc
Y L K L C Q E I K D K R L K L
586 tttaaggattattttgtggacgagaggaagaagcttgaaagcata
F K D Y F V D E R K K L E S I
631 aagagtgtagataacaacagcttgaagtgcgccatagatcatatc
K S V D N N S L K C A I D H I
676 atagaagctcaggaaaaaggagaaatcaacgaggataacgttctt
I E A Q E K G E I N E D N V L
721 tacattgttgaaaacataaatgttgctgcaattgaaacaacacta
Y I V E N I N V A A I E T T L
766 tggtcaattgaatggggcattgcggaactagttaataaccctgaa
W S I E W G I A E L V N N P E
811 atccagaagaagttgaggcatgagttggacactatgctcggggtc
I Q K K L R H E L D T M L G V
856 ggagttcagatctgtgagccagacgttcagaagctcccttacctt
G V Q I C E P D V Q K L P Y L

901 caagctgtgatcaaagagactcttcgattcagaatggccattcct
Q A V I K E T L R F R M A I P
946 cttttagtccctcacatgaaccttcatgaagcgaagcttgcaggt
L L V P H M N L H E A K L A G
991 tatgacatcccggcagagagcaagatcttggtcaatgcatggtgg
Y D I P A E S K I L V N A W W
1036 cttgccaacaatcccgctcactggaaaaacccaaatgagtttagg 1080
L A N N P A H W K N P N E F R

**Figure S6:** Nucleic acid and deduced amino acid sequence of *AgC3H* gene.

```
  1 ttggctaaagaagtgttgaaggaaaatgatcagcagttggctgac
    L  A  K  E  V  L  K  E  N  D  Q  Q  L  A  D
 46 aggcataggaacaaagctactgatatatttagtagaggtgggagt
    R  H  R  N  K  A  T  D  I  F  S  R  G  G  S
 91 gatttgatatgggctgattatgggcctcattatgttaaggtcagg
    D  L  I  W  A  D  Y  G  P  H  Y  V  K  V  R
136 aaagtttgtaatgttgagctttttactcccaagagacttgaagct
    K  V  C  N  V  E  L  F  T  P  K  R  L  E  A
181 ttgaggcccattagagaagatgaagttactgctatggttgagtcc
    L  R  P  I  R  E  D  E  V  T  A  M  V  E  S
226 attttcaaggattgcactgcttctgataagcagggaaagagtttg
    I  F  K  D  C  T  A  S  D  K  Q  G  K  S  L
271 ttgctaaggccttacttaggatcagtagcatttaacaacattaca
    L  L  R  P  Y  L  G  S  V  A  F  N  N  I  T
316 agactgtcatttggaaaaagatttgtcaactcagaaggtggaatt
    R  L  S  F  G  K  R  F  V  N  S  E  G  G  I
361 gatgagcaaggacaagagttcaagggaattgtttctaatggtatc
    D  E  Q  G  Q  E  F  K  G  I  V  S  N  G  I
406 aagatcggtgccaaagtgttcatgggagagtatgtgccatggctt
    K  I  G  A  K  V  F  M  G  E  Y  V  P  W  L
451 cgctggatgtttgctggagcaaacgatgtactcaaccagcacgaa
    R  W  M  F  A  G  A  N  D  V  L  N  Q  H  E
496 gctcgtcgggcacgcctcaccaaacagatcatggcagaacatact
    A  R  R  A  R  L  T  K  Q  I  M  A  E  H  T
541 cttgcacgcaacaaaaccgggggtgccaaagatcattttgttgat
    L  A  R  N  K  T  G  G  A  K  D  H  F  V  D
586 gcattgctcactcttcagaaacagtatgatttaagtgacgacact
    A  L  L  T  L  Q  K  Q  Y  D  L  S  D  D  T
631 gttatcactctcctatgggacatgatcactgcaggaatggacaca
    V  I  T  L  L  W  D  M  I  T  A  G  M  D  T
676 acttcaatctcagtggaatgggctatggctgagctagtcaagaac
    T  S  I  S  V  E  W  A  M  A  E  L  V  K  N
721 ccaagggtacagaagaaggcccaagaggagctggaccgggtaatc
    P  R  V  Q  K  K  A  Q  E  E  L  D  R  V  I
766 gggttggacaggatcatgactgaagctgacttctccaagctccct
    G  L  D  R  I  M  T  E  A  D  F  S  K  L  P
811 tacctgcaatgtgtagccaaggaagcactaagattgcaccctcct
    Y  L  Q  C  V  A  K  E  A  L  R  L  H  P  P
856 acccctatgatgctccctcacaaagccagtgccaataccaaactc
    T  P  M  M  L  P  H  K  A  S  A  N  T  K  L
```

901 ggaggctacgacatacctaaaggatccatcgtgcatgttaatgta

G G Y D I P K G S I V H V N V

946 tgggccattgcccgcgaccctgcattgtggaaagaccctctcgaa

W A I A R D P A L W K D P L E

991 ttttggcccgaaagattcctggaagaagatgttgacatgaaaggc

F W P E R F L E E D V D M K G

1036 cacgattatcgactactaccgtttggtgctggaaggagaatatgt

H D Y R L L P F G A G R R I C

1081 cctggtgctcagcttgctataaacttggtgacatctatgttggga

P G A Q L A I N L V T S M L G

1126 catcttttgcaccattttacttggactccaccggaaggtgttaag

H L L H H F T W T P P E G V K

1171 cctgaggaagttgacatgagcgagaaccctggaatggtg 1209

P E E V D M S E N P G M V

**Figure S7:** Nucleic acid and deduced amino acid sequence of *AgF5H* gene.

```
  1 atggaaaccaacactaccgccatgacaattctcttcttcattctt
    M  E  T  N  T  T  A  M  T  I  L  F  F  I  L
 46 ccgctgcttagtttctttctcttgtccagctttcgacgtaaacgt
    P  L  L  S  F  F  L  L  S  S  F  R  R  K  R
 91 taccctccaggcccgaaaggttggcccatcatcggcaacttgttg
    Y  P  P  G  P  K  G  W  P  I  I  G  N  L  L
136 atgatggacaagctatctcatcgtggactggctaaacttgctgct
    M  M  D  K  L  S  H  R  G  L  A  K  L  A  A
181 caatatggcggccttgtccacctccgtatgggttttcttcacatg
    Q  Y  G  G  L  V  H  L  R  M  G  F  L  H  M
226 ttcaccgtttcgactcccgatatggcccgagaagttcttcaaatt
    F  T  V  S  T  P  D  M  A  R  E  V  L  Q  I
271 caagacaacatttttgccaaccgtcctgctaccatgaatattagc
    Q  D  N  I  F  A  N  R  P  A  T  M  N  I  S
316 tacttaacttatgaccgagcggatatggctttcgcaaattacggg
    Y  L  T  Y  D  R  A  D  M  A  F  A  N  Y  G
361 ccgttttggcgccaaatgcggaaaatatcggtcatgaagttattt
    P  F  W  R  Q  M  R  K  I  S  V  M  K  L  F
406 agccgtaaaagggcagagtcgtgggactctgtccgtgaagaggtt
    S  R  K  R  A  E  S  W  D  S  V  R  E  E  V
451 gatgacatggtgaaaatcgtgttgtcgaagacggggtgttcggtt
    D  D  M  V  K  I  V  L  S  K  T  G  C  S  V
496 aatattggagagcttgtgttcgggttaactaggaacattatttat
    N  I  G  E  L  V  F  G  L  T  R  N  I  I  Y
541 cgggcagctttcgggacgttgtcgcacgaaggccaagacgagttt
    R  A  A  F  G  T  L  S  H  E  G  Q  D  E  F
586 atcaagatattgcaggagttttcgaaactgtttggtgcattcaat
    I  K  I  L  Q  E  F  S  K  L  F  G  A  F  N
631 atctgtgattttgttccgggattaacttgggcagatccgcaaggg
    I  C  D  F  V  P  G  L  T  W  A  D  P  Q  G
676 ttcatgggtcgggtggttaaagctagagcatcgcttgatggattc
    F  M  G  R  V  V  K  A  R  A  S  L  D  G  F
721 atagactcaataatagatgcacacattgaaaaaaagaagagcagt
    I  D  S  I  I  D  A  H  I  E  K  K  K  S  S
766 aaaaatggtattatcgacgagggaaacagtgatatggtgtatgaa
    K  N  G  I  I  D  E  G  N  S  D  M  V  Y  E
811 ttgctggatttttacggtgaagaaaaggctaaagtcagcgagttt
    L  L  D  F  Y  G  E  E  K  A  K  V  S  E  F
856 gaagatcagaacagctccttgaagctcacaagagataacatcaaa
    E  D  Q  N  S  S  L  K  L  T  R  D  N  I  K
```

901 gccattatcatggatgtaatgtttggtgggacggagacggtagca
A I I M D V M F G G T E T V A
946 tctgcaatagagtgggccatgtcagagctaatgaggagcccaaaa
S A I E W A M S E L M R S P K
991 gacctcaaaaaagtccaacaagaactcgtcaatgttgttgggctt
D L K K V Q Q E L V N V V G L
1036 caccgtcgtgttgaagaaagtgatttcgacaagctcacttacctc
H R R V E E S D F D K L T Y L
1081 aaatgctgcataaaagagactcttagactccaccctcccatccca
K C C I K E T L R L H P P I P
1126 ctacttttacacgagacggcccaagatgcggaggttgctggatat
L L L H E T A Q D A E V A G Y
1171 cacattccggcaaggtctcgagtcatcataaactcatgggccatc
H I P A R S R V I I N S W A I
1216 aacagagacccaaactcgtggactgacccggacacattcaagcct
N R D P N S W T D P D T F K P
1261 tctaggtttctacaagagggtatgcctgactttaaaggaagcaac
S R F L Q E G M P D F K G S N
1306 tttgagttcataccatttgggtcgggtcggaggtcttgtccaggc
F E F I P F G S G R R S C P G
1351 atgcaacttggactttatgcacttgagatagctgtggctcacctt
M Q L G L Y A L E I A V A H L
1396 ctgcattgttttaactgggagttacctgatggtatgaagccaagt
L H C F N W E L P D G M K P S
1441 gaagttgatactgatgatgtgtttggtctcactgctccgagggcg
E V D T D D V F G L T A P R A
1486 actcgacttgtggctgtgccaactccacgcttgttgtgtcctatc
T R L V A V P T P R L L C P I
1531 tcctga 1536
S *

**Figure S8:** Nucleic acid and deduced amino acid sequence of *Ag4CL* gene.

```
  1 atggaaacagttgacccgagaaatggctactccggcgcaacaaaa
    M  E  T  V  D  P  R  N  G  Y  S  G  A  T  K
 46 acattccagagtatccggcccacagtcccattaccagcagaaaaa
    T  F  Q  S  I  R  P  T  V  P  L  P  A  E  K
 91 acatgtctgtcggcctcagcatactctctccctcaaagccaac
    T  C  L  S  A  S  A  Y  S  L  S  L  K  A  N
136 agccgatggcctgacgcaacagcaatcatagactcccacacaggc
    S  R  W  P  D  A  T  A  I  I  D  S  H  T  G
181 caccgagtatcctactcggagtttactcgactcactcaaaacctg
    H  R  V  S  Y  S  E  F  T  R  L  T  Q  N  L
226 gcctccttttaactactcatctcaaactcaccattggtcaaacc
    A  S  F  L  T  T  H  L  K  L  T  I  G  Q  T
271 gccttcatcctatcccccaactccacttccattcccattctctat
    A  F  I  L  S  P  N  S  T  S  I  P  I  L  Y
316 ttctcacttctctctattggtgtcatcatctctcctgctaatcct
    F  S  L  L  S  I  G  V  I  I  S  P  A  N  P
361 gtctccacaatttccgatatttcccgccaaattcacttatccaat
    V  S  T  I  S  D  I  S  R  Q  I  H  L  S  N
406 cctgtcattgcttttgccacgtccgccacgtttcataagcttcca
    P  V  I  A  F  A  T  S  A  T  F  H  K  L  P
451 actctccactacccaaccatactcctcgactcgcccgagtttgag
    T  L  H  Y  P  T  I  L  L  D  S  P  E  F  E
496 tcaatgatgctgcagcagcagcagcagcagactcgtagttttagt
    S  M  M  L  Q  Q  Q  Q  Q  Q  T  R  S  F  S
541 ttgagttatcaagtgagtcagtctgatactgctgcggtgctctac
    L  S  Y  Q  V  S  Q  S  D  T  A  A  V  L  Y
586 tcgtcgggaacaacgggtcaggtcaaaggcgtggagttgactcat
    S  S  G  T  T  G  Q  V  K  G  V  E  L  T  H
631 cgcaatttgattgcgattacttctagttatcaggtgaataataat
    R  N  L  I  A  I  T  S  S  Y  Q  V  N  N  N
676 ttaagggagactccgtctgttgtcatgtacactgccccgtttttt
    L  R  E  T  P  S  V  V  M  Y  T  A  P  F  F
721 catgtcattggatttttttactgtatcaagtctgtgagtttaagc
    H  V  I  G  F  F  Y  C  I  K  S  V  S  L  S
766 gaaactatagtggtgatgaataggtttgacttgaagaagatgtgt
    E  T  I  V  V  M  N  R  F  D  L  K  K  M  C
811 agagctgtgcaagactacaaggtcacgcagattgtgggagcgcca
    R  A  V  Q  D  Y  K  V  T  Q  I  V  G  A  P
856 ccggtggttgtggcaatgattaaggatagtgccacggctgaattt
    P  V  V  V  A  M  I  K  D  S  A  T  A  E  F
```

901 aatctcgagtcgttggagatggtgggatccgggggtgctcccctg

N L E S L E M V G S G G A P L

946 ggaaaggatgtcattgctaccttcaaagccaaatttccggataaa

G K D V I A T F K A K F P D K

991 gaactgtttcagggttatggaatgactgaaacagccggagccatc

E L F Q G Y G M T E T A G A I

1036 tttcgatctacaagcccggaagaaagtttgcgctggggttcggta

F R S T S P E E S L R W G S V

1081 gggaaacttacaggacactgtgaggcaagaattgttgacccagag

G K L T G H C E A R I V D P E

1126 tcaggaaacgctctgccacctggcaagctaggggagctatggatt

S G N A L P P G K L G E L W I

1171 agaggacctctagttatgaaaggctatattggtaatgctcaggca

R G P L V M K G Y I G N A Q A

1216 acttctgaaacattggtaggtgacgggtggttaagaacgggagat

T S E T L V G D G W L R T G D

1261 ctttgttacatcgacaaagaaggttttcttttcgttgttgacaga

L C Y I D K E G F L F V V D R

1306 ctgaaggaattgataaagtacaagggataccaggtccctcctgca

L K E L I K Y K G Y Q V P P A

1351 gagcttgaacaattgcttcagtcccatccagagataatagatgct

E L E Q L L Q S H P E I I D A

1396 gcagttattccattccctgatgaagaggctggggaagtgcctatg

A V I P F P D E E A G E V P M

1441 gcatgcgtggttaaacattcggaaagtaggatcactgagtcacta

A C V V K H S E S R I T E S L

1486 gtgatggattttattgcaaagcaggttgcgccgtacaagaaaata

V M D F I A K Q V A P Y K K I

1531 aggcgggtatggttcgttacttcaattccaaaaagcgcagctggg

R R V W F V T S I P K S A A G

1576 aaaatattgaggaaggacttaaggaaggctgctgctcaaggatct

K I L R K D L R K A A A Q G S

1621 ctctccaagctgtga 1635

L S K L *

**Figure S9**: Nucleic acid and deduced amino acid sequence of *AgHCT* gene.

```
  1 atgaagatcaccgtgaaagaatcaacgttagtgcctccggccgag
    M  K  I  T  V  K  E  S  T  L  V  P  P  A  E
 46 gccacgccacgacggagtctctggaatgctaacgtagacctcgtc
    A  T  P  R  R  S  L  W  N  A  N  V  D  L  V
 91 gtaccgaattttcacacacctagcgtgtatttttacaggccaaat
    V  P  N  F  H  T  P  S  V  Y  F  Y  R  P  N
136 ggtagtgacaaatttttgacacaaaagtacttaaggatgcattg
    G  S  D  K  F  F  D  T  K  V  L  K  D  A  L
181 agtagagctttggttcctttttatcccatggctggacgattaaaa
    S  R  A  L  V  P  F  Y  P  M  A  G  R  L  K
226 agagacgatgatggtcgcgtcgagattgattgtaatggagaaggc
    R  D  D  D  G  R  V  E  I  D  C  N  G  E  G
271 gtgctcttcgttgaggctgagtcggacggtgtggtggatgatttt
    V  L  F  V  E  A  E  S  D  G  V  V  D  D  F
316 ggtgactttgctcccacgttagagctccgccaactgattccgacg
    G  D  F  A  P  T  L  E  L  R  Q  L  I  P  T
361 gttgattattcacttgggatatcatcgtattcgttgttggttttg
    V  D  Y  S  L  G  I  S  S  Y  S  L  L  V  L
406 cagataacctttttcaaatgtggtggagtctcactaggtgttggt
    Q  I  T  F  F  K  C  G  G  V  S  L  G  V  G
451 atgcaacaccatgctgcagatggagcctctggactccatttcatc
    M  Q  H  H  A  A  D  G  A  S  G  L  H  F  I
496 aacacatggtccgatatggctcgcggtcttgacctcaccctcgca
    N  T  W  S  D  M  A  R  G  L  D  L  T  L  A
541 ccattcatagaccgcaccctcctccgtgctcgtgagccgcctcaa
    P  F  I  D  R  T  L  L  R  A  R  E  P  P  Q
586 cctgcatttccccacattgaataccaaccgcctccttccatgaag
    P  A  F  P  H  I  E  Y  Q  P  P  P  S  M  K
631 tccaaccctgatccgacaattattcccgaaacatccgtctcaatc
    S  N  P  D  P  T  I  I  P  E  T  S  V  S  I
676 tttaaaattactcgagaacagctcaatgctctcaaagccaagtcc
    F  K  I  T  R  E  Q  L  N  A  L  K  A  K  S
721 aaggaagatggaaacacagtggcttatagctcctatgaaatgctg
    K  E  D  G  N  T  V  A  Y  S  S  Y  E  M  L
766 gcaggacacatgtggcggactgtgtgtaaagcccgtggacttgct
    A  G  H  M  W  R  T  V  C  K  A  R  G  L  A
811 gatgatcaagaaagcaagctgttcatcgcaactgatggaaggttc
    D  D  Q  E  S  K  L  F  I  A  T  D  G  R  F
856 aggttacttcctccactcccaccaggttactttggcaatgtaatt
    R  L  L  P  P  L  P  P  G  Y  F  G  N  V  I
```

901 ttcactaccacgcctatagctgcagcaggtgatctcatatcaaag
F T T T P I A A A G D L I S K
946 ccactgtggtatagcgctagtagaattcatgatgcattggcacgg
P L W Y S A S R I H D A L A R
991 atggacaatgattatctaaggtcagcgcttgactacctggaattg
M D N D Y L R S A L D Y L E L
1036 cagcctgatcttaaagcacttgttcgcggggcacattctttcaag
Q P D L K A L V R G A H S F K
1081 tgccccaaccttggaattactagctgggctagattgcctatacac
C P N L G I T S W A R L P I H
1126 gacgctgattttggatggggaaggccaatattcatgggacctgga
D A D F G W G R P I F M G P G
1171 ggaattgcttaa 1182
G I A *

**Figure S10:** Nucleic acid and deduced amino acid sequence of *AgCOMT* gene.

1 atggcgcatatcaagagtactgcatctgaagcagaagaagcttgt

M A H I K S T A S E A E E A C

46 ttgtttgcattgcaactcgcaactgcttctgtacttcccatgact

L F A L Q L A T A S V L P M T

91 ctgaaagtggccattgagcttgaccttttggagatcattgctaat

L K V A I E L D L L E I I A N

136 gctggtccgggagcgtacgtaactcccagtgagctagcctccatg

A G P G A Y V T P S E L A S M

181 ctgcccacatcaaaccttgatgcagcgcctatgctcgaccgtata

L P T S N L D A A P M L D R I

226 ctcagaatcctggctagctacttgaagtggatatgtcataactgg

L R I L A S Y L K W I C H N W

271 agtgatgaagagtgcttgagattcctgacaaattgttaccaaaca

S D E E C L R F L T N C Y Q T

316 ctcgcagatgagggaaaggtagttgttgcagagtctattcttccg

L A D E G K V V V A E S I L P

361 gagcaacctgaaacttcacttgttactaagactgtccttcagtta

E Q P E T S L V T K T V L Q L

406 gacgcctatatgttgatagatattcctggtggaagagaaaggact

D A Y M L I D I P G G R E R T

451 gaaaaagagtttgaggcattagctaaacgtgcaggattcaaacgt

E K E F E A L A K R A G F K R

496 ttcaacaagctgtgctgtgcttttaatatttggattatggaattg

F N K L C C A F N I W I M E L

541 tgcaagtag 549

C K *

**Figure S11:** Nucleic acid and deduced amino acid sequence of *AgCCoAOMT* gene.

```
  1 atggcttctaatgctgaatccaaacattcagaagttgggcacaag
    M  A  S  N  A  E  S  K  H  S  E  V  G  H  K
 46 agtcttttgcagagtgatgctctttgtcagtatatacttgaaaca
    S  L  L  Q  S  D  A  L  C  Q  Y  I  L  E  T
 91 agtgtgtacccaagagagccagaggcaatgaaagagcttagagat
    S  V  Y  P  R  E  P  E  A  M  K  E  L  R  D
136 gtcactgccaagcatccatggaatctgatgacaacatcagctgat
    V  T  A  K  H  P  W  N  L  M  T  T  S  A  D
181 gaagggcagttcttgagcatgcttttgaagctcatcaatgccaag
    E  G  Q  F  L  S  M  L  L  K  L  I  N  A  K
226 aacaccatggagattggtgtttacactggttattctctccttgcc
    N  T  M  E  I  G  V  Y  T  G  Y  S  L  L  A
271 acggccctggctcttccagatgatgggaagattttggcattggac
    T  A  L  A  L  P  D  D  G  K  I  L  A  L  D
316 atcaacagagaaaactatgaaattggattaccaattattgaaaaa
    I  N  R  E  N  Y  E  I  G  L  P  I  I  E  K
361 gctggagttggtcacaaaattgacttcagagagggccctgctttg
    A  G  V  G  H  K  I  D  F  R  E  G  P  A  L
406 cctgttcttgatcatatgcttgaagatgggaagtttcatgggaca
    P  V  L  D  H  M  L  E  D  G  K  F  H  G  T
451 ttggattttgtattcgttgatgctgacaaagataactatatcaac
    L  D  F  V  F  V  D  A  D  K  D  N  Y  I  N
496 taccacaagagattaattgatttagtgaaaatcggaggacttatc
    Y  H  K  R  L  I  D  L  V  K  I  G  G  L  I
541 ggctacgacaacaccctttggaatggttctgtggcacagccagct
    G  Y  D  N  T  L  W  N  G  S  V  A  Q  P  A
586 gatgctcccatgaggaagtatgtaaggtactacagagactttgtg
    D  A  P  M  R  K  Y  V  R  Y  Y  R  D  F  V
631 atcgagcttaacaaagccctggctgctgatcccaggattgagatc
    I  E  L  N  K  A  L  A  A  D  P  R  I  E  I
676 tgcatgcttcctgttggtgatggagttaccctgtgccgtcgtatc
    C  M  L  P  V  G  D  G  V  T  L  C  R  R  I
721 agctga 726
    S  *
```

**Figure S12:** Nucleic acid and deduced amino acid sequence of *AgCCR* gene.

```
  1 atgggtattgtccgaaccgatgaacatcgccaatcggaaattgaa
    M  G  I  V  R  T  D  E  H  R  Q  S  E  I  E
 46 agtttccggcgacttcttgtttcttgcgccggagcttgtaaaagt
    S  F  R  R  L  L  V  S  C  A  G  A  C  K  S
 91 aaagaagagcaagacaccggaaaaaatcatgtcatgaacgttgac
    K  E  E  Q  D  T  G  K  N  H  V  M  N  V  D
136 gacgatgtggtgcaggataaggtggtgtgcgttaccagcggcgta
    D  D  V  V  Q  D  K  V  V  C  V  T  S  G  V
181 tcttacctgggaattgctattgtcaaccagctattaatccgtgga
    S  Y  L  G  I  A  I  V  N  Q  L  L  I  R  G
226 tataacgttcgtattatcgttgataatcaagatgatgtagagaat
    Y  N  V  R  I  I  V  D  N  Q  D  D  V  E  N
271 ctaagggagatggaaacatctggagagatgaggggaacgaataac
    L  R  E  M  E  T  S  G  E  M  R  G  T  N  N
316 aggattggaattgtcatggccaaactatatgaagctgagagttta
    R  I  G  I  V  M  A  K  L  Y  E  A  E  S  L
361 tctgacgtatttagtggttgctgcggagttttccacacttcagct
    S  D  V  F  S  G  C  C  G  V  F  H  T  S  A
406 tttattgatcctgctgggctttctggctattctaaatttatggct
    F  I  D  P  A  G  L  S  G  Y  S  K  F  M  A
451 gatttagaggtgaaggcagcacagaatgtaatcgaggcatgtgca
    D  L  E  V  K  A  A  Q  N  V  I  E  A  C  A
496 gcaacaccttcaataagacattgcgtgcttacgtcttcacttttg
    A  T  P  S  I  R  H  C  V  L  T  S  S  L  L
541 gcttgtacatggaaagattactcactcgacaatgtatctcctgtg
    A  C  T  W  K  D  Y  S  L  D  N  V  S  P  V
586 attaatcatatttgctggagtgatgaatccctttgtatagagaga
    I  N  H  I  C  W  S  D  E  S  L  C  I  E  R
631 aagctttggtatgctttgggcaagctgagggcagaaaaggctgcc
    K  L  W  Y  A  L  G  K  L  R  A  E  K  A  A
676 tggagagtagccgaggagaggggacttaagctggccacaatctgc
    W  R  V  A  E  E  R  G  L  K  L  A  T  I  C
721 ccaggccttattaaaggttctgaattctttaacagaaatacaaca
    P  G  L  I  K  G  S  E  F  F  N  R  N  T  T
766 tcaacgattgcttatttaaaaggtgctcaagaaatgtatgaaaga
    S  T  I  A  Y  L  K  G  A  Q  E  M  Y  E  R
811 ggactgttggcaactgttgatgtaaatagattagcggaggcgcat
    G  L  L  A  T  V  D  V  N  R  L  A  E  A  H
856 gtatgtgtgtttgaggaattgaacaggacagcgtccggaaggtat
    V  C  V  F  E  E  L  N  R  T  A  S  G  R  Y
```

901 atctgcttcgaccaagtgatcaagaacgaggaagaggcggagacg
I C F D Q V I K N E E E A E T
946 ctggctcaggagacagggatacacataaatgtgatatctgaaact
L A Q E T G I H I N V I S E T
991 gaaaatacatctggcaatgctcctacacagcttgaactctcaaat
E N T S G N A P T Q L E L S N
1036 ttaaaactttctaggttgatgtcaagagttagaaattgtaagaag
L K L S R L M S R V R N C K K
1081 atcttttag 1089
I F *

**Figure S13:** Nucleic acid and deduced amino acid sequence of *AgCAD* gene.

```
  1 atgagcaagacggtgtgcgtaacgggagcatcaggctacatagca
    M  S  K  T  V  C  V  T  G  A  S  G  Y  I  A
 46 tcatggcttgtcaagtttttgcttcaacgtggttacaccgttaaa
    S  W  L  V  K  F  L  L  Q  R  G  Y  T  V  K
 91 gcttctgtcagagatcccaatgatcctaagaaaactgctcacttg
    A  S  V  R  D  P  N  D  P  K  K  T  A  H  L
136 cttgcacttgagggagccaaggatagacttcagctgtttaaagca
    L  A  L  E  G  A  K  D  R  L  Q  L  F  K  A
181 aatctactggaagaagggtcctttgatgctgcggttactggttgt
    N  L  L  E  E  G  S  F  D  A  A  V  T  G  C
226 gaaggtgtcttccatacagcatcaccttttactcatgctgttgat
    E  G  V  F  H  T  A  S  P  F  T  H  A  V  D
271 gatccacaggcagaattgatcgatcctgcggtcaagggcacactt
    D  P  Q  A  E  L  I  D  P  A  V  K  G  T  L
316 aatgtactcgggtcatgtgcaaaaactccctcggtaaaaagagtg
    N  V  L  G  S  C  A  K  T  P  S  V  K  R  V
361 gttttgacttcttctgttgctgcggttgcatacaatggtaagcct
    V  L  T  S  S  V  A  A  V  A  Y  N  G  K  P
406 aggacccctgatgtcgtagtggatgagtcatggttttcagaccca
    R  T  P  D  V  V  V  D  E  S  W  F  S  D  P
451 gagttttgcaaggaaaataagatgtggtatgttctttcaaagacg
    E  F  C  K  E  N  K  M  W  Y  V  L  S  K  T
496 ttagctgaagatgcagcatggaagtttgtcggagaaaaaggcatc
    L  A  E  D  A  A  W  K  F  V  G  E  K  G  I
541 gacatggttacaatcaatccagcaatggttatcggccctcttctg
    D  M  V  T  I  N  P  A  M  V  I  G  P  L  L
586 caaccaaccctgaacacaagtgcagctgcaattttgaacctaata
    Q  P  T  L  N  T  S  A  A  A  I  L  N  L  I
631 aatggagctcagacatatccaaatgcttccttcggttgggttaat
    N  G  A  Q  T  Y  P  N  A  S  F  G  W  V  N
676 gtcaaggatgttgctaatgctcatattctagcatatgaaactcct
    V  K  D  V  A  N  A  H  I  L  A  Y  E  T  P
721 tccgctaatggaagatactgtttagttgagagtgttgtgcaccat
    S  A  N  G  R  Y  C  L  V  E  S  V  V  H  H
766 tcaggggttgtcgatattctgcgcaagctatatccttctcttcaa
    S  G  V  V  D  I  L  R  K  L  Y  P  S  L  Q
811 ctaccagacaagtgtgcagacgacaaaccctttacaccaacatat
    L  P  D  K  C  A  D  D  K  P  F  T  P  T  Y
856 caggtttccaaggacaaaacaaaaagcttgggcatcagctacatt
    Q  V  S  K  D  K  T  K  S  L  G  I  S  Y  I
```

901 ctgctagaggatggtatcaaggaaaccgtcgagagcttgaaagat

L L E D G I K E T V E S L K D

946 aagaaatttttttctgtctaa 966

K K F F S V *

**Figure S14:** Nucleic acid and deduced amino acid sequence of *AgPOD* gene.

```
  1 atgctggctgtgagtattactttcagctgttttcttgggttttca
    M  L  A  V  S  I  T  F  S  C  F  L  G  F  S
 46 caaggccagcttatgtttggtttttactctaattcttgcccaaat
    Q  G  Q  L  M  F  G  F  Y  S  N  S  C  P  N
 91 gctgaatcaattgtttccagagttgtcagtgataaagtcagagaa
    A  E  S  I  V  S  R  V  V  S  D  K  V  R  E
136 agcccaaataatgttcctattttgctcaggcttcactttcatgat
    S  P  N  N  V  P  I  L  L  R  L  H  F  H  D
181 tgttatgttcaggggtgtgatgcatcaatactgattgacaatggc
    C  Y  V  Q  G  C  D  A  S  I  L  I  D  N  G
226 ccaaacgcggagaaaactgcgtttgggcaccaaggagttagaggt
    P  N  A  E  K  T  A  F  G  H  Q  G  V  R  G
271 tttgatgtaattgaagcagccaaggctcaattggaaagtgtttgc
    F  D  V  I  E  A  A  K  A  Q  L  E  S  V  C
316 cctggtgtagtttcttgtgctgacattgttgccatggctgcaaga
    P  G  V  V  S  C  A  D  I  V  A  M  A  A  R
361 gatgctgttgctttggcgaatggtccgagatatcaagtgcaaact
    D  A  V  A  L  A  N  G  P  R  Y  Q  V  Q  T
406 ggtagaagagatggaatagtttcggataaatctctagcagaggat
    G  R  R  D  G  I  V  S  D  K  S  L  A  E  D
451 atgccggatgttaatgattcaattcagactctcaaggccaaattt
    M  P  D  V  N  D  S  I  Q  T  L  K  A  K  F
496 gttagcaagggactcaatgataaagaccttgtggttcttagtgct
    V  S  K  G  L  N  D  K  D  L  V  V  L  S  A
541 gcacatacaatcggcacaacagcatgcttcttcatggaaaaaaga
    A  H  T  I  G  T  T  A  C  F  F  M  E  K  R
586 ctatacagctttttcccaaatggtggtggttctgatccgtcaata
    L  Y  S  F  F  P  N  G  G  G  S  D  P  S  I
631 aaccccgctttcctaccagaattacaggccacgtgtccgcgtaat
    N  P  A  F  L  P  E  L  Q  A  T  C  P  R  N
676 ggagatgtcaacgtccggctaccaatggaccgcggcagtgaacag
    G  D  V  N  V  R  L  P  M  D  R  G  S  E  Q
721 actttcgacaagcacattttgcagaacatcagaaccggttttgca
    T  F  D  K  H  I  L  Q  N  I  R  T  G  F  A
766 gtcatagcatctgatgcaagtttatatgatgatgttatgacaagg
    V  I  A  S  D  A  S  L  Y  D  D  V  M  T  R
811 agcgtagttgactcgtatttcggcccccctaaatccggttcttgga
    S  V  V  D  S  Y  F  G  P  L  N  P  V  L  G
856 ccgtctttcgagacagattttgttaactccatgataaagatgggt
    P  S  F  E  T  D  F  V  N  S  M  I  K  M  G
```

901 agtattgatgttaagaccggtactgaagggcaaatcaggcgcgtt
S I D V K T G T E G Q I R R V
946 tgtagagctttcaattaa 963
C R A F N *
